# From Insulin Resistance to Mitochondrial Failure: A Staged Model of Impaired Metabolic Flexibility in Schizophrenia

**DOI:** 10.3390/biom16101397

**Published:** 2026-09-24

**Authors:** Myles Cubitt, Bart Ellenbroek, Anne M. Haase

**Affiliations:** 1 School of Psychological Sciences, Victoria University of Wellington, Wellington 6012, New Zealand; 2 School of Health, Victoria University of Wellington, Wellington 6012, New Zealand; anne.haase@vuw.ac.nz

**Keywords:** schizophrenia, insulin resistance, mitochondria, PI3K/AKT, mTOR, depression, bipolar disorder

## Abstract

Schizophrenia is a highly heritable neuropsychiatric disorder whose pathophysiology remains poorly understood. While neurotransmitter, neurodevelopmental, synaptic, and dysconnectivity hypotheses explain important aspects of the disorder, growing evidence suggests that metabolic dysfunction also plays a central role. This narrative review synthesises evidence from genetics, cell signalling, molecular neuroscience, and metabolism to propose a theoretical framework linking insulin resistance and mitochondrial dysfunction to schizophrenia. We argue that genetic susceptibility and environmental risk factors converge to produce insulin resistance, impaired insulin signalling, and chronic metabolic stress. Reduced insulin signalling suppresses PI3K/AKT activity, while nutrient overload initially hyperactivates mTORC1 independently of PI3K/AKT. Together, these abnormalities shift cellular metabolism away from oxidative metabolism, promote neuroinflammation, impair mitophagy, disrupt mitochondrial dynamics, and increase oxidative stress. Progressive mitochondrial dysfunction subsequently reduces ATP production and downregulates mTORC1, resulting in persistent bioenergetic impairment. We propose that these metabolic abnormalities provide mechanistic links to hallmark features of schizophrenia, including GABAergic and dopaminergic dysfunction, abnormal neurodevelopment, synaptic pathology, cortical structural changes, and disrupted brain connectivity. Finally, we suggest that schizophrenia differs from mood disorders through greater prenatal neurodevelopmental disruption and reduced mitochondrial repair capacity, producing more severe and persistent metabolic dysfunction.

## 1. Introduction

Schizophrenia is a severe mental illness that affects roughly 0.62% of the world’s population [1]. The disorder consists of positive symptoms (hallucinations and delusions), negative symptoms (affective flattening, alogia, avolition/apathy, anhedonia/asociality, and attention impairment), and disorganised symptoms (disordered thoughts and bizarre behaviour) [2]. Schizophrenia symptoms are often debilitating, leading to significant disability, early mortality, and economic burden [2,3]. Schizophrenia was first coined by Professor Eugen Bleuler in 1908, when he argued against Kraepelin’s view that the disorder was a premature form of dementia and suggested that schizophrenia is “not a disease in the strict sense but appears to be a group of diseases” [4]. Modern evidence supports Bleuler’s initial conceptualisation, suggesting that the schizophrenia spectrum has a complex, multifactorial aetiology involving common and rare genetic variants, epigenetic processes, and a wide range of environmental factors [5]. Despite over a century of research, the pathophysiology of schizophrenia is still not clear; remissions are rare, and current antipsychotic medications typically only produce good outcomes in positive symptoms and have less of an impact on negative and disorganised symptoms [2].

Several major theories have been proposed to explain the pathophysiology of schizophrenia, reflecting the disorder’s complex and multifactorial nature. The dopamine hypothesis was an early model that remains largely influential, proposing that increased presynaptic dopamine synthesis and release within striatal pathways contribute to psychotic symptoms [6]. However, accumulating evidence suggests that dopamine dysregulation may represent a downstream consequence of broader neurobiological disturbances. The glutamate hypothesis proposes that hypofunction of N-methyl-D-aspartate (NMDA) receptors disrupts excitatory neurotransmission, causes cortical disinhibition, and leads to secondary dopamine abnormalities [7]. Complementing these neurotransmitter-based models, the neurodevelopmental hypothesis argues that genetic vulnerability and environmental insults during critical periods of brain development produce latent abnormalities that manifest clinically during adolescence or early adulthood [8]. The dysconnectivity hypothesis suggests that schizophrenia arises from aberrant functional and structural connectivity between brain regions, resulting in impaired integration of information across large-scale neural networks involved in cognition, perception, and executive functioning [9]. Similarly, the synaptic hypothesis proposes that excessive synaptic pruning, loss of synaptic connectivity, and synaptic plasticity, particularly during adolescence, contribute to disease onset and progression [10].

More recently, research on genetic and environmental factors for schizophrenia has been pointing towards the disorder having abnormal glucose metabolism and energy production in both the periphery and the brain [11]. Similar trends have been observed in inflammation, insulin resistance (IR), and insulin signalling, suggesting they play a significant role in the pathophysiology of schizophrenia [12,13]. Of note is a recent review by Sarnyai and Ben-Shachar [14], which proposes a framework that redefines schizophrenia as a disease that progresses due to ongoing bioenergetic failure and suggests that schizophrenia is a disorder of impaired dynamic metabolic flexibility, where impaired brain fuel selection impacts brain function and disease progression over time.

The purpose of this review is to build on the framework set by Sarnyai and Ben-Shachar [14], expanding it to cellular signalling mechanisms that produce this metabolic inflexibility, while also incorporating findings surrounding insulin resistance, insulin signalling, the PI3K/AKT/mTOR pathway, and mitochondrial function. Furthermore, the review aims to examine where these mechanistic pathways converge with leading theories of schizophrenia development, with the hope of providing a unifying framework.

## 2. Materials and Methods

Given the broad and interdisciplinary nature of the topic, a narrative review approach was adopted to integrate evidence from psychiatry, neuroscience, endocrinology, metabolism, and molecular biology. Consistent with narrative review methodology, the literature searches were iterative. The literature was identified through searches of PubMed, Scopus, and Google Scholar. Scopus was included to broaden coverage beyond biomedical journals indexed in PubMed and to capture the interdisciplinary literature relevant to metabolism, mitochondrial biology, and molecular signalling. The initial literature search used combinations of terms including “schizophrenia”, “diabetes”, “heritability/genetics”, “insulin signalling”, “insulin resistance/hyperglyc(a)emia”, “PI3K/AKT”, “mTOR”, “mitochondria”, “metabolism”, “metabolic syndrome”, “glycolysis”, and “oxidative phosphorylation”. Priority was given to direct human studies on schizophrenia, as well as to meta-analyses, systematic reviews, and mechanistic investigations. However, as metabolism and mitochondria are emerging and under-researched in schizophrenia, animal and cellular studies, along with converging evidence from other disease populations, were included when they provided evidence relevant to the proposed biological mechanisms and where research in schizophrenia populations was lacking. Further searches were informed by mechanistic findings implicating related biological pathways, following key citations from the initial literature search and by using broader terms in PubMed, Scopus, and Google Scholar. This approach allowed exploration of biological processes not identified in the initial search strategy, but which appeared potentially relevant to the proposed mechanistic framework.

## 3. Schizophrenia-Spectrum, Heritability, and Genetic Overlap

Research suggests that schizophrenia-spectrum disorders are highly heritable and suggests a genetic or familial relationship between each disorder on the spectrum. Heritability estimates based on twin study data are around 60–80% for schizophrenia, schizoaffective disorder, and bipolar disorder [15,16,17,18,19]. Twin studies show substantial genetic overlap between schizoaffective disorder and both schizophrenia (0.77) and mania (0.88), with model-fitting suggesting that all genetic influences on schizoaffective disorder are shared with the other two disorders [20]. Genome-wide association study (GWAS) shows a significant genetic overlap between schizophrenia and schizotypy, further suggesting that the schizophrenia-spectrum disorders are driven by similar polymorphisms in all phenotypes [21]. Interestingly, research suggests a significant genetic overlap between bipolar disorder and schizophrenia, which have historically been thought to be etiologically separate disorders [22]. Cross-disorder risks between schizophrenia and bipolar disorder are lower than within; however, model-fitting still suggested genetic correlations between 0.46 and 0.68 [20,23]. Although these disorders are highly heritable and have significant genetic overlap, there is not a 100% phenotype expression rate, indicating that relatives of people with schizophrenia may carry latent genetic susceptibility [24]. Nonetheless, evidence suggests that each disorder has shared genetic vulnerabilities. The question then arises: what do these shared genes encode, and how do they contribute to psychological dysfunction?

## 4. Insulin Resistance and Insulin Signalling

Individuals with schizophrenia often have co-occurring insulin resistance, prediabetes, and type 2 diabetes. A meta-analysis examining 17–57 studies by Vancampfort et al. [25] found that diabetes was prevalent in 9.2% of bipolar patients, 11.8% of schizophrenia-spectrum patients, and 11.5% of schizophrenia only patients. Early evidence indicates that insulin resistance may have a much larger prevalence in schizophrenia, with several studies using the homeostasis model assessment of insulin resistance (HOMA-IR) showing a prevalence rate of 30–60% [26,27,28]. It has been well established that treatment with antipsychotic medications may have an impact on insulin resistance, metabolic syndrome, and type 2 diabetes, with many suggesting that these metabolic abnormalities are strictly due to antipsychotic medication [29]. However, abnormal glucose metabolism has been observed in schizophrenia before the development of antipsychotic drugs [30,31,32,33]. Similarly, a more recent systematic review and meta-analysis done by Pillinger et al. [34] examined glucose homeostasis in first-episode (FEP) schizophrenia across 15 studies (731 patients, 614 controls). Patients showed significantly elevated fasting glucose, fasting insulin, and HOMA-IR compared with controls. These differences persisted after excluding antipsychotic-treated participants and after controlling for factors such as BMI, ethnicity, diet, and exercise. These findings suggest that insulin resistance and likely impaired insulin signalling are present early in schizophrenia and may suggest they are not solely attributable to antipsychotic treatment or lifestyle factors.

### 4.1. Genetic Influences

Perhaps unsurprisingly, several studies have identified shared genetic risk factors between type 2 diabetes, metabolic syndrome, mood disorders and schizophrenia [35,36]. Of particular interest is the MIR137 gene, which is a leading contender for schizophrenia development [37,38]. MIR137 encodes a short non-coding microRNA that regulates the expression of other genes [39]. Genome-wide RNA profiling of a human neuronal cell line following miR-137 manipulation identified four interesting risk loci affected by miR-137: CANCA1C, MAD1L1, TCF4 and BDNF [40]. The CACNA1C region encodes pancreatic islet (L-type) voltage-dependent calcium channels (VDCCs) and has been reported as a risk gene for schizophrenia in several studies [37]. VDCCs play a complex role in insulin resistance, with their function varying by tissue. In pancreatic tissue, dysfunction of VDCCs impairs glucose-stimulated insulin secretion, contributing to the development of type 2 diabetes and leading to hyperglycaemia [41,42].

Several GWAS indicate that the MAD1L1 (mitotic arrest deficient-like 1, MAD1) region and the BDNF region (brain-derived neurotrophic factor) reach significance in schizophrenia [37,40,43,44]. MAD1 regulates the spindle assembly checkpoint during mitosis and is expressed in the basal ganglia, the ventral tegmental area, and the hippocampus [45]. MAD1’s role in spindle assembly causes downstream neuronal polarity, ensuring proper neuronal migration and differentiation [46]. Additionally, MAD1 activates MAD2, which then interacts with other mitotic regulators, forming a module that regulates insulin receptor endocytosis in cells [47]. Similarly, BDNF activates transduction cascades, including the IRS1/2/PI3K/AKT (AKT is also known as PKB) pathway, which is involved in neuronal survival, growth, and plasticity and serves as a modulator of neurotransmission [48]. Activation of this pathway is also essential for the survival of beta cells that produce and secrete insulin [49]. Taken together, these findings suggest that genetic abnormalities in CACNCA1C, MAD1L1, and BDNF may dysregulate neuronal development, survival, growth, and plasticity, while also causing systemic insulin resistance, impaired insulin secretion, and destruction of beta cells that produce insulin.

Given that the genetic abnormalities could cause neuronal abnormalities, it may seem as if insulin resistance and reductions in insulin secretion are just a comorbid side effect of the genes that are associated with increased risk for schizophrenia. However, evidence suggests that insulin signalling is essential for the development and maintenance of the central nervous system [50].

### 4.2. Insulin Signalling

Historically, insulin was thought to be incapable of crossing the blood–brain barrier (BBB) and thus was considered not to affect the central nervous system [50]. In the 1990s, insulin was discovered in the brain, and insulin receptors are expressed throughout the brain, with increased expression in the olfactory system, hypothalamus, cerebral cortex, hippocampus, and pituitary gland. Insulin is considered a neurotrophic factor, and its signalling is involved in several aspects of the CNS, including proliferation and survival, synaptic plasticity and connectivity, synaptogenesis, and glial cell development [50,51].

#### 4.2.1. Neurotrophic Growth Factors and the PI3K/AKT Pathway

Insulin also directly and indirectly affects other neurotrophic factors, such as IGF-1, nerve growth factor (NGF), and BDNF, which play significant roles in the CNS by promoting neuronal survival and development from embryonic stages through to adulthood, promoting neurite outgrowth, regenerating damaged neurons, and supporting neuronal function [52,53]. Insulin stimulates the liver’s production of IGF-1 by co-regulating the growth hormone/IGF-1 axis [54]. IGF-1 is also suggested to affect insulin sensitivity by binding to insulin receptors with low affinity, thereby enhancing insulin’s effectiveness and improving glucose metabolism [55]. Insulin signalling may also regulate BDNF. Takach et al. [56] found that exendin-4, an anti-diabetes drug, restored regular BDNF transport in hippocampal neurons by stimulating glucagon-like peptide 1 to activate the insulin signalling pathway. Similar results have been observed in mouse models, in which metformin (an anti-insulin-resistance drug) enhances BDNF expression and exerts neuroprotective effects by activating the PI3K/AKT pathway [57]. However, the side effects of insulin resistance are likely to play a more significant role in the dysfunction of BDNF and NGF, as discussed in later sections. Nonetheless, evidence suggests that insulin signalling is directly involved and has bidirectional relationships in the maintenance of several neurotrophic factors.

As mentioned earlier, BDNF activates the PI3K/AKT pathway. Insulin and IGF-1 also activate this signalling cascade and the MAPK/ERK pathway (also known as the RAS-RAF-MEK-ERK pathway). The PI3K/AKT pathway promotes neuronal survival, whereas the MAPK pathway promotes cellular death; however, there is significant crosstalk between the pathways, which further complicates the signalling cascade [58]. Insulin binds its receptor, thereby activating the downstream insulin receptor substrate (IRS) adaptor protein. The IRS then activates PI3K, which in turn activates PKB/AKT. PKB/AKT then deactivates several proteins that promote apoptosis, including BAD, glycogen synthase kinase 3 (GSK3) and the transcription factor FOXO. GSK3 inactivation then inhibits tau phosphorylation, and FOXO inactivation prevents FASL and BIM activation. PKB/AKT inactivates BAD through phosphorylation and activates mTOR through phosphorylation [59,60]. Similarly, activation of the insulin receptor also activates another adaptor protein, SHC, which then recruits growth factor receptor-binding protein 2 (GRB2) and son of sevenless (SOS) [50]. GRB2 and SOS then activate RAS, which leads to activation of RAF, MEK and MAP kinase proteins (MAPK) ERK1/2. Crosstalk between IRS1 and GRB2 leads to an alternative pathway by which insulin activates ERK1/2. In contrast, crosstalk between PKB/AKT and RAF leads to RAF dephosphorylation, ultimately resulting in PI3K/PKB-mediated inhibition of ERK1/2. See Figure 1 for a detailed diagram of PI3K/AKT signalling under normal and insulin-resistant conditions.

PI3K/AKT, MAPK, and associated pathways have been directly and indirectly related to several psychological disorders, including depressive disorders and schizophrenia [61,62,63,64,65]. Drug-naïve patients with schizophrenia have been shown to have lower PI3K serum levels than controls [66]. Furthermore, reductions in AKT expression have been observed in schizophrenic brain samples, and reductions in AKT phosphorylation have been observed in several different types of schizophrenia neuronal samples [67,68]. Similarly, abnormalities in MAPK phosphorylation and expression have been observed in several regions of the brain in patients with schizophrenia [69,70,71]. However, the association between these pathways and psychiatric illness is likely due to the function of their downstream target enzymes and proteins.

#### 4.2.2. Neurotransmitter Function

PI3K/AKT phosphorylates the type A γ-aminobutyric acid receptor (GABA_A_R) [72]. Phosphorylation of GABA_A_R increases the number of GABA_A_R receptors on the plasma membrane, thereby enhancing receptor-mediated synaptic transmission in neurons and, in turn, synaptic plasticity. Postmortem schizophrenic brain samples have shown reduced GABA_A_R protein and mRNA, and antipsychotic-free patients with schizophrenia have lower GABA_A_R levels in the hippocampus [73]. GABA_A_ receptors are the primary target for the inhibitory neurotransmitter GABA [74]. GABA binding to its receptors hyperpolarises the neuron, ultimately suppressing excitability in the central nervous system. GABA neurons in the ventral tegmental area (VTA) inhibit dopamine release, as well as exert dopamine-independent effects [75].

Insulin signalling may also contribute to dopamine function. Current evidence suggests that insulin affects dopamine differently depending on the site of administration: insulin applied to the striatum increases dopamine release, whereas insulin applied to the VTA suppresses release by inhibiting excitatory inputs (for a full review, see Gruber et al. [76]). The VTA modulates the midbrain dopamine system, acting as a homeostatic controller of dopamine release into the striatum, and mouse models suggest insulin induces long-term depression (LTD) of the VTA through endocannabinoid signalling [77,78]. Interestingly, models of schizophrenia and human imaging studies have shown increased VTA neuronal activity, which may drive excess dopamine synthesis capacity and release in the striatum (nigrostriatal and mesolimbic pathways) [79]. Taken together, the evidence may suggest that reduced insulin signalling increases dopamine production in the striatum in two ways. First, by reducing GABA inhibition in the VTA; second, by reducing LTD in the VTA through the endocannabinoid pathway.

In summary, insulin-mediated PI3K/AKT phosphorylation of GABA_A_R receptors may cause GABAergic dysfunction, which has been associated with several schizophrenic traits, including positive symptoms, impaired cognition, emotion, motor processing, and sensory processing [80]. Similarly, abnormal GABA function and insulin signalling in the VTA may disrupt dopamine signalling in the nigrostriatal and mesolimbic pathways, which have long been associated with positive symptoms of schizophrenia, psychosis, and several aspects of cognition [81]. However, a reduction in insulin signalling is only one way that insulin resistance may be involved in schizophrenia pathophysiology; hyperglycaemia may produce several other mechanisms involved in the development of schizophrenia.

### 4.3. Insulin Resistance (Hyperglycaemia)

Insulin resistance and resulting hyperglycaemia (elevated blood glucose) lead to nutrient overload, hypoxia, and the accumulation of degraded or unfolded proteins [82]. These metabolic abnormalities then cause a cascade of abnormal effects, including but not limited to oxidative stress, inflammation, apoptosis, and several downstream changes to metabolism [82,83,84,85,86,87]. Upon further inspection, all these changes that occur in type 2 diabetes due to insulin resistance are also implicated in schizophrenia [88].

#### 4.3.1. Neurotrophic Growth Factors

As previously mentioned, insulin resistance and hyperglycaemia may play a more significant role in regulating NGF and BDNF than insulin signalling itself. One study by Zhu et al. [89] showed that high glucose levels increase NGF and BDNF expression in Schwann cells in vitro, suggesting that glucose or metabolic processes may regulate NGF and BDNF in the nervous system. Similar results have been seen in NGF levels in diabetics; however, the evidence is unclear, with some studies showing an increase in NGF, while others show a downregulation of NGF, typically in the periphery [90,91,92,93,94]. Despite conflicting evidence, a recent review by Massimino et al. [95] suggested that the balance between mature NGF and its precursor, proNGF, is disrupted, thereby reducing NGF signalling. Hyperglycaemia increases reactive oxygen species (ROS) through abnormal mitochondrial function and enzyme activity [96]. Similarly, glucose concentrations have been negatively correlated with antioxidant defence enzymes in patients with type 2 diabetes [97]. Peroxynitrite is an ROS that forms in diabetes and impairs metalloproteinase production, which cleaves proNGF into its mature form, thereby reducing NGF levels [98]. Both increased ROS and decreased antioxidant defence have been regularly associated with schizophrenia, which may suggest that the physiology of schizophrenia is the perfect environment for developing super ROS, such as peroxynitrite, and thus may suggest a potential mechanism for the downregulation of NGF associated with schizophrenia [99,100,101,102,103].

Glutamate’s role in GABAergic neurons may also be another hyperglycaemia-mediated mechanism that downregulates NGF. Evidence from animal models and cellular lines shows that GABAergic neurons are the primary producers of NGF in the cortex, hippocampus, striatum, and basal forebrain [104]. Glutamate can inhibit developing GABA neurons at both presynaptic and postsynaptic sites and sustained high glutamate levels act as a negative feedback loop that counteracts hyperexcitability by downregulating GABA receptors [105,106]. In vivo human and mouse models, and in vitro cellular models of diabetes, suggest that hyperglycaemia alters glutamate signalling and increases glutamate in the brain [107,108,109,110,111,112,113]. Similarly, GABAergic neurons have been suggested to act as glucose sensors, altering their function in response to glucose availability [114]. Abnormal glutamate signalling and increased levels of glutamate have been found in the brains of schizophrenia patients [115]. Taken together, the evidence suggests that impaired metalloproteinase enzymes and glutamate functioning may be one mechanism by which NGF is downregulated in the development of schizophrenia. However, if there is a lack of glucose in the brain due to downregulation of GLUT1 transporters, other mechanisms may contribute to a continuous reduction in NGF across the trajectory of schizophrenia. Dysregulation of GABA neurons via the PI3K/AKT pathway may also contribute to the downregulation of NGF in the central nervous system (CNS). Theoretically, initial decreases in NGF during schizophrenia development could be due to hyperglycaemia-mediated ROS production and abnormal glutamate signalling, which are then sustained by reduced insulin-mediated PI3K/AKT phosphorylation of GABA after glucose transport into the brain has been reduced.

Studies examining BDNF in diabetics paint a clearer picture, with plasma BDNF levels being lower in people with type 2 diabetes, and higher fasting glucose levels predicting lower BDNF levels in the brain [116,117]. BDNF is the most prominent neurotrophic factor and is expressed throughout the CNS, particularly in the neocortex, amygdala, hypothalamus, and hippocampus [118]. BDNF release is primarily regulated by glutamate neuronal activity and Ca^2+^-dependent signalling [119]. The endoplasmic reticulum (ER) in neurons extends from the nucleus right out to the axons and dendrites and is the primary store of calcium in the brain [120]. Ca^2+^ release from the ER is required for cognitive function and several other critical physiological functions, but Ca^2+^ depletion leads to further ER stress; thus, the ER is highly regulated and is constantly debating whether to release its Ca^2+^ stores [121]. Interestingly, hyperglycaemia in cardiovascular cells induces ER stress, leading to the unfolded protein response, ROS, and ultimately apoptosis [122]. No studies have examined the direct mechanistic metabolic outcomes of hyperglycaemia on ER and Ca^2+^ in human neurons; however, several studies suggest that reduced insulin signalling induces Ca^2+^ dysregulation in animal neuronal cells [123]. Rat models indicate that high glucose abnormally increases intracellular Ca^2+^ in neurons and microglia via activation of purinergic (P2) receptors, potentially representing an initial step in Ca^2+^ dysregulation [124]. Another potential mechanism is that misfolded proteins associated with hyperglycaemia induce ER stress, thereby further promoting Ca^2+^ release [125,126]. Taken together, the evidence suggests that initial hyperglycaemia promotes Ca^2+^ release and protein misfolding, thereby amplifying ER stress and further promoting Ca_2+_ release, ultimately disrupting the balance between intracellular and extracellular Ca^2+^.

A final mechanism by which Ca^2+^ is regulated in the ER when other mechanisms fail is the sarcoplasmic/endoplasmic reticulum Ca^2+^-ATPase (SERCA) pump [121]. Insulin resistance has been associated with reduced SERCA pump activity and abundance [127,128,129]. This reduction is likely due to increased ROS, as SERCA pumps are susceptible to oxidative modifications and high levels of peroxynitrite are associated with their downregulation [121,130]. Similarly, hypoxia may be another factor associated with SERCA dysfunction. Hypoxia occurs when cellular oxygen consumption exceeds oxygen delivery and is a known complication of diabetes and hyperglycaemia [131]. HIFs are transcription factors that regulate the hypoxic response by activating genes and indirectly repressing other transcription factors and microRNAs [132]. Under hypoxic conditions, HIF-1α upregulates SERCA2b expression, thereby maintaining calcium homeostasis and preventing cell death [133]. However, hypoxia-inducible factor (HIF)-mediated responses to hypoxia are also impaired in neurons and several other tissues of diabetics [131]. Current evidence clearly suggests that this impaired HIF response results from hyperglycaemia disrupting HIF-1α stability and function [134]. There are several suggestions for how hyperglycaemia dysregulates HIF-1α, including lowering NO levels via ROS, repressing expression, and promoting proline hydroxylase domain (PHD) mediated degradation and ubiquitin–proteasomal activity [131]. However, evidence for these mechanisms is often conflicting, possibly due to differences in function across cell types [134].

HIF-1α plays several roles beyond upregulating SERCA2b expression; it is involved in angiogenesis (blood vessel formation) and erythropoiesis (red blood cell production), and controls mitochondrial metabolism, thereby reducing oxidative stress, preventing cell death and inflammation [135]. Several systematic reviews and meta-analyses show that schizophrenia is associated with vascular abnormalities, reductions in blood flow, hypoxia, and metabolic changes related to hypoxia, such as increased lactate levels and lower (acidic) pH, which may implicate abnormal HIF signalling [136,137,138,139,140]. Older neurodevelopmental models of schizophrenia have proposed that foetal/prenatal hypoxia interacts with genetic susceptibility, and many established risk genes appear to be regulated by hypoxia and HIF-1α signalling, including AKT1 and BDNF [141]. More recent research using mRNA pathway analysis has indicated that several genes associated with schizophrenia are involved in the HIF-1α signalling pathway and oxidative metabolism [142,143]. However, most evidence comes from genetic or epidemiological studies; direct research on HIF abundance and activity in human samples from patients with schizophrenia is currently lacking [135]. Given that hyperglycaemia may degrade HIF in a PHD-mediated manner, relying solely on mRNA studies may indicate that hypoxia signalling cascades are involved in schizophrenia, but suggest little about how HIF functions after expression [144]. Nonetheless, evidence of HIF functionality in schizophrenia may be seen in HIF-1α’s downstream transcriptional targets.

HIF-1α coordinates adaptation to hypoxia by inducing angiogenic signalling through VEGF expression and promotes metabolic reprogramming through increased expression of glucose transporters (GLUT1 & GLUT3) and glycolytic enzymes [145,146]. VEGF stimulates both vasculogenesis and angiogenesis; it is produced in various cell types and then transported into other cells experiencing hypoxia [147,148]. Given the hypoxia and hypoxia-associated markers associated with schizophrenia, we may expect to see a clear pattern of increased VEGF, GLUT1 and GLUT3 expression. One meta-analysis and systematic review by Misiak et al. [149], which included 15 studies (982 patients and 791 controls), found that VEGF was significantly increased in multiple episodes of schizophrenia and remained the same as controls in first-episode psychosis. These differing results may indicate that VEGF, and thus HIF-1α activity, changes over disease progression. However, the multiple episode schizophrenia group was all medicated, and several of the drug-naive patients in the included studies found drug treatment rapidly increased VEGF. This is consistent with other research in animal models showing common antipsychotic treatments increase VEGF and angiogenesis in a time-dependent manner, suggesting antipsychotic treatment may be a confounding factor [150,151].

On the other hand, GLUT1 and GLUT3 transporter expression paints a clearer picture, with studies showing neuron-specific reductions in mRNA expression in the dorsolateral prefrontal cortex of schizophrenia patients [152,153]. Similar results have been observed for the glycolytic enzymes regulated by HIF, with patients showing decreased expression of hexokinase, phosphofructokinase, and glucophosphoisomerase [152,154]. Overall, these findings may suggest that HIF-1α signalling is disrupted in schizophrenia, despite the hypoxic conditions and increased expression of HIF-1α regularly associated with the condition. Furthermore, this hyperglycaemia-mediated dysfunction of HIF-1α signalling may lead to dysregulation of SERCA pumps. Animal models of schizophrenia and psychosis support this theory, showing dysregulation of SERCA pumps and genes related to their production; however, research has not directly assessed SERCA levels in human schizophrenia patients or samples [155,156].

Despite this lack of direct evidence for schizophrenia, converging evidence from diabetic models suggests that initial hyperglycaemia and hyperglycaemia-associated abnormalities in glutamate- and GABA-mediated neuronal function increase neuronal calcium influx, thereby dysregulating ER Ca^2+^ homeostasis and reducing BDNF production. This reduction in CA^2+^ and BDNF may then be sustained by the hypoxic and ROS-producing environment created by hyperglycaemia, through dysregulation of HIF and SERCA pumps.

#### 4.3.2. Metabolism, Inflammation and mTORs Role

mTOR dysfunction is another factor associated with both diabetes and schizophrenia [157,158,159]. There are two main mTOR complexes, mTORC1 and mTORC2. mTORC1’s role is to act as a sensor, responding to intracellular and environmental stressors (growth factors, DNA damage, oxygen levels, glucose, lipids, amino acids, and ATP levels) and to balance between anabolism and catabolism by activating growth processes and suppressing cell death [157,160]. On the other hand, mTORC2 is activated by growth factors and is a key regulatory molecule that is involved in several of the processes mentioned above. mTORC2’s defining role is its ability to phosphorylate AGC family kinases (AKT, PKC, SGK), fully activating them and thus acting as a signal amplifier and selector rather than a cellular sensor that directly activates signalling cascades [161]. However, the role of mTOR in the progression of schizophrenia is likely complex, as evidence from diabetes suggests that hyperglycaemia leads to upregulation of mTORC1 and downregulation of mTORC2, whereas schizophrenia postmortem studies suggest downregulation of both mTOR subunits [162,163,164]. However, several theoretical mechanisms suggest that mTOR function changes in response to metabolic stress and energy source, which may be involved throughout schizophrenia’s developmental trajectory, resulting in two key mechanistic shifts in metabolism.

Under normal conditions, glucose is the brain’s primary energy source [165]. Most of the glucose is processed in the mitochondria of neurons through oxidative phosphorylation to produce ATP, and only a small amount (10–12%) is metabolised through aerobic glycolysis (AG) [166,167]. AG is the process by which glucose is converted to lactate, and it typically occurs in astrocytes [168]. Glycolysis in the brain is a multistep metabolic pathway in which glucose is converted to pyruvate by several key enzymes, including hexokinase (HK1/2), phosphofructokinase-1 (PFK-1), glyceraldehyde-3-phosphate dehydrogenase (GAPDH), and pyruvate kinase (PK and PKM2 in astrocytes) [168,169,170]. PK is typically the final step of glycolysis, producing ATP and pyruvate, which is then suggested to be transported from the astrocyte to the neuron for conversion into acetyl-CoA by pyruvate dehydrogenase for metabolism via oxidative phosphorylation, generating 32 ATP molecules [171]. However, under low-oxygen conditions (anaerobic) or high activity (aerobic), pyruvate and NADH are converted by lactate dehydrogenase (LDH) into lactate and NAD+, producing only 2 ATP molecules, but regenerating NAD+ and allowing glycolysis to continue [172].

#### 4.3.3. Initial Metabolic Shift

As mentioned previously, hyperglycaemia induces a wide range of cellular stressors and is therefore likely to significantly affect mTORC1 function in multiple ways. Nutrient overload (excess glucose, fatty acids, and amino acids) is a factor associated with hyperglycaemia and leads to hyperactivation of mTORC1 [82,160,173]. High energy availability enables cells to produce ample ATP via glycolysis and oxidative phosphorylation, resulting in a high ATP-toADP ratio [174]. This high ATP-to-ADP ratio inactivates AMPK, another key energy sensor. Under normal conditions, activated AMPK phosphorylates the TSC complex, which acts as a GTPase-activating protein (GAP) for another protein called Rheb, converting Rheb into its inactive form and ultimately inhibiting mTORC1 [174]. Thus, under high ATP-to-ADP ratios, inactive AMPK prevents activation of TSC1/2, thereby maintaining constant Rheb signalling and ultimately driving mTORC1 activation. Similarly, chronic circulating insulin seen in insulin resistance cannot activate PI3K/AKT, but still activates the MEK/ERK pathway, which inhibits TSC1/2, allowing Rheb to stay active [175,176]. Finally, amino acid overload associated with hyperglycaemia contributes to mTORC1 hyperactivation, as amino acids activate Rag GTPases, which then translocate mTORC1 to the lysosome, allowing full activation to occur [177,178]. In summary, nutrient overload puts the brakes on TSC1/2 activation through AMPK, inhibits its activity through MEK/ERK, and translocates mTORC1 to the lysosome, allowing mTORC1 to remain active whilst the PI3K/AKT pathway is suppressed (see Figure 2).

Activation of mTORC1 by nutrient overload further increases insulin resistance. It does so through sustained downstream activation of S6K1; S6K1 activation, in turn, leads to inhibitory phosphorylation of IRS1 [179]. IRS1 is an insulin receptor substrate protein, a central cytoplasmic adaptor molecule involved in the transfer of extracellular signals from receptor proteins; therefore, its inhibition prevents insulin signalling [180]. IRS1’s activation recruits PI3K to the membrane and activates it. Thus, hyperglycaemia induces mTORC1 hyperactivation, which further increases insulin resistance. Furthermore, this mechanism is another way the PI3K/AKT pathway could be downregulated by inhibiting IRS1’s ability to recruit and activate PI3K after growth factor signalling. This mechanism, alongside reduced growth factor production, is likely driving the findings that individuals with diabetes have hyperactive mTORC1 and hypoactive mTORC2, as inhibition of IRS1’s ability to recruit and activate PI3K would prevent mTORC2 activation via the PI3K/AKT pathway. Several studies support this theory, having shown that downregulation of insulin–PI3K signalling results in mTORC2 inactivation [181,182,183].

Interestingly, mTORC1 has also been suggested to shift glucose metabolism from oxidative phosphorylation to glycolysis [160]. This metabolic shift leads to aerobic glycolysis, increasing glucose uptake and lactate production, a phenomenon originally termed the Warburg effect in cancer research, though the same shift occurs in activated immune cells (discussed in later sections) [184]. mTORC1 facilitates this shift by inducing and regulating the transcription factors HIF-1α and Myc, which upregulate the expression and activity of enzymes and proteins involved in glycolysis [185]. However, as noted earlier, evidence indicates that hyperglycaemia disrupts HIF-1α stability and function [134]. Therefore, under chronic hyperglycaemia and associated hypoxia, we may observe mTORC1-mediated upregulation of HIF-1α production but disrupted function, whereas Myc would be upregulated and remain functional. Currently, evidence for a direct Myc mechanism in neurons or schizophrenia patients is limited, and the detailed mechanisms by which Myc coordinate glycolytic enzymes have been characterised predominantly in cancer cell models; caution is warranted when extrapolating these mechanisms directly to neurons or glia without cell-type-specific validation.

As discussed earlier, HIF-1α has been characterised in normal cellular conditions and regulates key enzymes and GLUT1/3, which have been found to be decreased in schizophrenia populations, including approximately 20% reductions in GLUT1 and GLUT3 transcripts in pyramidal neurons [145,146,153,154]. GLUT1 is primarily expressed in the endothelial cells of the blood–brain barrier and astrocytes (controlling glucose uptake into the parenchyma), whereas the high-affinity GLUT3 primarily controls glucose uptake by neurons [186]. Under hyperglycaemic and hypoxic conditions, mTORC1 would be unable to upregulate HIF-1α, leading to decreased overall brain glucose and neuronal glucose availability and increased reliance on the lactate transporter MCT1, which has been found to be increased in schizophrenia [14].

Importantly, much of the evidence for altered GLUT1/3, MCT1, and lactate metabolism in schizophrenia comes from postmortem tissues or from individuals with chronic established illness, which may point towards the cellular environment and hypometabolism at the later stages of the disease, but suggests little about disease progression. During hyperglycaemic clamps, brain glucose increases in healthy individuals, but this response is progressively blunted (although there is still a rise) in people with obesity and type 2 diabetes [187]. Diabetic animal models show cerebral glucose utilisation and lactate production initially increasing, followed by a decrease in GLUT1 expression occurring after more chronic exposure [188]. A recent meta-analysis by Townsend, Pillinger, Selvaggi, Veronese, Turkheimer and Howes [11] found that absolute frontal glucose metabolism was significantly lower in chronic schizophrenia (g = −1.18) than in first-episode patients, whose frontal metabolism did not differ meaningfully from healthy controls (g = −0.09). These findings may indicate a time-dependent progression towards hypometabolism and may suggest that metabolic dysfunction is dynamic across prodromal, first episode, and chronic schizophrenia.

A relevant consideration is an emerging theory in the diabetes literature suggesting that neurons may resist glycolytic flux, independent of mTORC1 pathways and activity. Glycolytic flux, in this theory, is dependent on hexokinase 2 (HK2), an enzyme that controls the first step of glycolysis and is susceptible to glucose-driven protein stabilisation and unscheduled glycolysis under conditions of nutrient excess [189]. HK2 is highly expressed in peripheral Schwann cells and in CNS microglia but is very low in neurons and astrocytes [190,191,192]. Consequently, although both peripheral and CNS neurons experience increased glucose uptake during diabetes, CNS neurons are not susceptible to HK2-dependent glycolytic overload [193].

Consistent with this theory of neuronal and astrocyte resistance to glycolytic overload, evidence from the prodromal period points not to an emerging glycolytic phenotype, but to early signs of inflammatory and insulin-related dysregulation. Studies have identified peripheral inflammatory, oxidative, metabolic, and hormonal profiles distinguishing clinical high-risk (CHR) individuals who later develop psychosis from those who do not [194]. In a longitudinal FEP/CHR/control cohort, fasting insulin and insulin resistance worsened specifically in CHR individuals who converted to psychosis, independent of antipsychotic exposure [195]. Direct glycolytic or mitochondrial evidence in CHR remains limited: one antipsychotic-naïve cohort found lactate and mitochondrial complex activity comparable to controls, while another found elevated serum glucose alongside reduced lactate in at-risk individuals [196,197]. This pattern extends to first episode, with direct evidence implicating central insulin resistance. Gene expression in antipsychotic-naïve FEP patients overlaps with rodent models of central insulin resistance [198]. CSF studies similarly report elevated glucose with reduced lactate and pH in FEP, drug-naïve schizophrenia [199].

Overall, this evidence may suggest that initial prodromal and first-episode schizophrenia consists of a period of insulin resistance and underutilisation of glucose, potentially starting an initial cascade of later metabolic abnormalities. However, longitudinal evidence of this occurring in at-risk populations is currently lacking, and this mechanistic outline remains largely theoretical. In the following sections, we outline theoretical mechanisms by which inflammation arises during these periods, and how these early abnormalities may give rise to the later metabolic shift.

#### 4.3.4. Immuno-Metabolism

In the last 20 years, there has been a significant growth in immunometabolism research, as metabolic pathways have been seen to regulate immune function [200]. Microglia are the main immune cells in the CNS. They are highly mobile, respond rapidly, and are involved in phagocytosis and inflammatory functions [201]. Microglia can shift their phenotype, including to two well-known subtypes, M1 or M2; chronic activation of M1 leads to the secretion of pro-inflammatory cytokines and neurotoxic molecules, whereas M2 produces anti-inflammatory cytokines and neurotrophic factors [202]. However, it must be noted that it is increasingly recognised that the M1/M2 framework describes extremes of a continuum rather than genuinely discrete microglial states and should be interpreted with this caveat in mind [203]. As mentioned previously, astrocytes’ main role is to provide nutritional support to neurons; however, they are also involved in neuroinflammation [204]. Like microglia, astrocytes have an A1 state that is pro-inflammatory and neurotoxic, and an A2 state that is neuroprotective [202]. Interestingly, the Warburg effect has been implicated as a key regulator of immunometabolic pathways (including in microglia), with inflammatory immune cells relying on increased glycolysis, whereas anti-inflammatory cells tend to rely on oxidative phosphorylation [205,206]. Microglia maintain an equilibrium with ROS and antioxidant defence, and their antioxidant defence systems have been shown to be significantly stronger than those of neurons and astrocytes [207,208,209]. Microglia and astrocytes (to a lesser extent) differ from neurons in that, under normal conditions, they rely on ROS as a secondary signalling pathway [209]. This ROS signalling, known as redox signalling, is a key mechanism that shifts microglia towards their pro- or anti-inflammatory subtypes [210].

Perhaps unsurprisingly, both mTOR and AMPK have been shown to regulate different aspects of the CNS immune response [211]. mTORC1 facilitates microglial shift towards glycolysis by regulating the transcription factors HIF-1α and Myc, thereby promoting glycolysis and producing the M1 inflammatory phenotype [212,213]. However, microglial cells in diabetic conditions maintain nuclear translocation of HIF-1α [214]. This sustained nuclear translocation may suggest that microglia’s adaptation to a high-ROS environment allows HIF-1α to escape prolyl hydroxylase domain (PHD) mediated hydroxylation and subsequent degradation under hyperglycaemia. However, evidence remains limited, and no direct studies have compared the effects of hyperglycaemia on HIF-1α across different brain tissues. Nonetheless, this M1 glycolysis-dependent state interacts with other inflammatory signals, such as NF-κB, which induces the production of pro-inflammatory cytokines, including NLRP3 inflammasome components, TNF-α, IL-18, and IL-1β [211]. On the other hand, active AMPK in macrophages and microglia promotes an M2 anti-inflammatory response by inhibiting NF-κB and reducing the production of inflammatory cytokines [215]. Under normal conditions, this switch to the anti-inflammatory M2 state has been suggested to occur through AMPK signalling low-energy status, which then suppresses mTORC1 via TSC2 and Raptor, while also activating mTORC2, PGC-1α, and SIRT3, which go on to produce and regulate new mitochondria (see Figure 3) [211].

PGC-1α is a transcription factor that regulates the expression of genes for oxidative phosphorylation and mitochondrial function, enhancing oxidative cellular respiration by increasing the expression of electron transport chain components and mitochondrial DNA synthesis [216]. Similarly, PGC-1α also controls antioxidant defence and reduces ROS accumulation by regulating the expression of superoxide dismutase 2 (SOD2) and uncoupling protein 2 (UCP2) [217,218]. SIRT3 expression is also upregulated by PGC-1α [219]. SIRT3 regulates metabolism by allowing pyruvate derived from glycolysis to enter the Krebs cycle, ensuring fatty acid oxidation, and promoting amino acid utilisation [220,221]. SIRT3 also promotes mitochondrial quality control and decreases ROS through direct and indirect activation of SIRT1, FOXO3, and OPA1 [221,222]. These signalling cascades have been suggested to help microglia meet the high energy demand of producing anti-inflammatory molecules such as TGF-β and IL-10, while also suppressing inflammatory cytokine production [211]. PGC-1α and SIRT3 also support mitochondrial health and antioxidant defence in neurons and astrocytes, preventing ROS accumulation and allowing normal oxidative metabolism [223,224]. Overall, this mTORC1 and AMPK signalling cascade is adaptive in microglia, allowing them to switch between pro- and anti-inflammatory states, whereas in astrocytes and neurons, the same pathway acts as a mitochondrial quality-control and antioxidant mechanism.

Under acute hyperglycaemia, we would expect a shift toward the M1 inflammatory phenotype due to mTORC1 hyperactivation. Evidence from diabetic studies supports this theory, with cellular and animal models, as well as postmortem studies, showing chronic low-grade neuroinflammation, upregulation of mTORC1-mediated pro-inflammatory pathways in microglia, and increased production of pro-inflammatory cytokines [214]. Similarly, microglia are activated in schizophrenia, including high-risk psychosis and schizophrenia samples that are naïve to antipsychotic treatment [225]. A very recent mouse model study by Yan et al. [226] may support this theory, showing that hyperactivation of mTOR suppresses autophagy, leading to sustained elevation of pro-inflammatory cytokines. Activated microglia and the pro-inflammatory cytokines, chemokines, and ROS they release disrupt endothelial tight junctions and extracellular matrix components, leading to BBB breakdown and allowing peripheral inflammatory molecules and cells to infiltrate the central nervous system, thereby exacerbating neuroinflammation in a second-order fashion [227]. Significant crosstalk among microglia, neurons, astrocytes, endothelial cells, and peripheral leukocytes exacerbates BBB dysfunction and is mediated by excessive ROS production from hyperglycaemia in diabetic populations [228,229]. 16% of individuals with first-episode psychosis and 20–30% of individuals with schizophrenia show increased BBB permeability, suggesting that it may play a role in the development of the disorder, or be a by-product of another dysfunction [230,231].

Although theoretical in parts, including converging evidence that has not been examined directly in schizophrenia, the current evidence may suggest that hyperactive mTORC1 and hypoxia due to insulin resistance lead to neuroinflammation in glial cells. In prodromal and FEP cohorts, this may partially explain increased inflammation, without the overall metabolic changes due to neurons’ resistance to increased glycolysis. However, an important mechanistic question remains: how does this metabolic shift continue when mTORC1 is consistently found to be downregulated in schizophrenia patients?

#### 4.3.5. Final Metabolic Shift

mTORC1 also plays a significant regulatory role in mitochondrial function [232]. One important role of mTORC1 is in mitophagy, which is thought to be the primary mechanism of mitochondrial quality control in neurons [233]. Stress-induced mitophagy occurs due to oxidative stress, energy deprivation, or DNA damage. Mitophagy helps cells maintain healthy mitochondrial numbers and function, including regulating energy metabolism, preventing ROS production, and preventing cell death [234]. UNC-51-like kinase 1 (ULK1) and autophagy-related proteins (ATG) form the ATG/ULK1 complex, which regulates mitophagy initiation [233]. PINK1 is a damage-sensing protein that probes mitochondria and activates Parkin [235]. Parkin then tags damaged mitochondria with ubiquitin, marking them for mitophagy. mTORC1 phosphorylates ULK1 and ATG, preventing the activation of the ATG/ULK1 complex, blocking the initial step of mitophagy, and inhibiting the activation of downstream autophagy factors [236]. Similarly, cellular models of chronic hyperactivation of mTORC1 (missing TSC1/2) show deficient mitophagy and indicate that mTORC1 signalling is essential not only for autophagy initiation, but also for recognition and targeting of damaged material, through decreased PINK1 expression and impaired Parkin translocation [237]. Overall, evidence suggests that hyperactivation of mTORC1 may reduce mitophagy, leading to an accumulation of damaged mitochondria.

Mitochondria cannot be created de novo; they are formed by the fusion and fission of existing mitochondria, with only new mitochondrial proteome components being transcribed and translated before being transported into mitochondrial subcompartments, which are then either fused or fissioned into new mitochondria [238]. mTORC1 is involved in mitochondrial biogenesis by mediating mitochondrial fusion and fission and by increasing the transcription and translation of key mitochondrial components. mTORC1 promotes translation of mitochondrial fission process protein 1 (MTFP1) by phosphorylating 4E-BP1 and relieving 4E-BP1-mediated repression of cap-dependent translation [232]. Furthermore, MTFP1 is associated with phosphorylation and mitochondrial recruitment of the pro-fission GTPase DRP1, providing a mechanism through which mTORC1 activity can promote mitochondrial fission [232]. Similarly, mTORC1 may regulate the mitofusion GTPase proteins MFN1/2 and OPA1, which are essential for outer and inner mitochondrial fusion, respectively [239]. However, the direct mechanisms by which mTORC1 regulates DRP1, MFN1/2, and OPA1 remain unclear. Nonetheless, mTORC1 suppression under nutrient deprivation promotes fusion and increases mitochondrial efficiency, suggesting that hyperactivation of mTORC1 may have the opposite effect [238]. Taken together, the evidence may suggest that hyperactive mTORC1 perturbs the fission–fusion balance, promoting increased fission rates (see Figure 4 below).

Interestingly, an accumulation of damaged and fragmented mitochondria caused by nutrient excess has also been suggested to shift metabolism away from oxidative phosphorylation [240]. It has been suggested that high levels of fragmentation and damaged mitochondria disrupt the inner membrane and cristae structure (folding of the inner mitochondrial membrane that increases surface area for oxidative phosphorylation), leading to impaired electron transport chain complexes, which cause proton leakage, decreased ATP production, increased ROS, and further DRP1 activation [239]. DRP1 activation further promotes mitochondrial fission, creating a positive feedback loop of oxidative stress. Several changes to mitochondrial function have been observed in schizophrenia, including SNPs in genes coding the mitochondrial proteome, increased DRP1 expression, decreased OPA1 expression, decreased expression and activity of electron transport chain complexes, smaller/fragmented mitochondria, and a reduced number of mitochondria in several different neurons and areas of the brain [241,242,243]. Microglia also exhibit reduced mitochondrial number, volume, and increased damage in schizophrenia samples [244].

mTORC1 may be downregulated through several mechanisms after mitochondria are damaged and metabolism has moved away from oxidative phosphorylation. As mentioned previously, nutrient overload hyperactivates mTORC1 by inhibiting AMPK activation, driven by a high ATP-to-ADP ratio, thereby shifting metabolism towards glycolysis [245]. However, under chronic aerobic glycolysis, when metabolism is uncoupled from mitochondrial oxidative phosphorylation, ATP production is reduced, leading to a high ADP-to-ATP ratio [246]. This switch to a high ADP-to-ATP ratio prevents AMPK inhibition, activates it, and enables it to phosphorylate the TSC complex, ultimately inhibiting mTORC1 [245]. Similarly, fragmented and dysfunctional mitochondria produce ROS due to electron leakage, thereby causing significant DNA damage [247]. DNA damage activates two proteins known as tumour suppressors in cancer research, p53 and p63 [248]. Activation of p53 and p63 leads to activation of sestrin1/2 and REDD1, respectively, both of which then inhibit mTORC1. Furthermore, chronic hyperactivation of mTORC1 and the associated downstream inhibition of IRS1, combined with reduced growth factor levels, drive downregulation of the PI3K/AKT pathway, thereby reducing AKT-mediated inhibition of the TSC1/2 complex [179]. Taken together, the evidence suggests that mTORC1 could be downregulated after mitochondrial damage through reactivation of AMPK, activation of the DNA damage pathway, and further reduction in AKT-mediated inhibition of TSC1/2.

This evidence may suggest that hyperactive mTORC1 further drives neurons, astrocytes, and microglia away from oxidative phosphorylation by damaging mitochondria and inhibiting mitophagy. Damage to mitochondria and reduced mitophagy then lead to excessive ROS production, mitochondrial DNA damage, and a significant reduction in ATP production, which paradoxically downregulates mTORC1. However, once mTORC1 activity is reduced, this may allow the resumption of mitophagy and mitochondrial fusion, leading to a shift towards oxidative phosphorylation in an AMPK-mediated fashion, similar to what has been suggested to occur under normal conditions in microglia [211]. Nonetheless, several mechanisms may prevent mitochondrial repair under high-ROS conditions, independent of mTOR function.

First, mitochondrial DNA lacks several DNA repair pathways found in other cells, so prolonged, sustained ROS-induced DNA damage may not be repaired [247]. Defective, DNA-damaged mitochondria would continue to produce ROS in a DRP1 feed-forward mechanism, and the damage may exceed fusion and mitophagy repair capacity, continuing the dysfunction in the oxidative phosphorylation respiratory chain [239,249]. Furthermore, PDIA1 (an enzyme involved in redox homeostasis and repair of oxidative damage) functions as a reductase for DRP1, maintaining normal mitochondrial function [250]. Interestingly, oxidative overload and stress cause dysfunction and inactivity of PDIA1, and mouse models with PDIA1 knocked out show mitochondrial fragmentation and increased ROS levels by increasing DRP1 activity, which may further explain how ROS interact with DRP1 to create a positive feedback loop [250,251]. Second, excessive ROS production from these damaged mitochondria may disrupt lysosomal membranes and inhibit lysosomal function by causing lipid peroxidation of lysosomal membranes, inactivating luminal enzymes, and impairing vascular ATPase-dependent acidification [252]. Lysosomes are particularly vulnerable to ROS due to their high iron and lipid content [253]. Chronic mitochondrial stress has also been shown to repress lysosomal biogenesis and cause dysfunction, blocking mitophagy [254]. Similar results have been observed in diabetic conditions, with mitochondrial ROS inducing lysosomal dysfunction, impairing autophagy, and producing M1 phenotype macrophages [255]. Finally, elevated ROS has been associated with reduced expression and activity of the mitofusion proteins MFN1/2 and OPA1, thereby preventing the fusion of newly formed healthy mitochondria [256]. Overall, there are several theoretical mechanisms by which mitochondrial damage could be sustained independently of mTORC1 in schizophrenia.

In brief, these mechanisms may suggest a two-phase, cell-type-dependent metabolic trajectory. During the initial phase (in prodromal, high-risk individuals), insulin resistance and nutrient overload may sustain mTORC1 activity, despite impaired PI3k/AKT signalling, promoting a shift towards glycolysis in glial (inflammatory) cells, where HK2-dependent glycolytic activity can support a pro-inflammatory phenotype. However, neurons may resist this glycolytic shift; instead, persistent insulin resistance and nutrient overload drive mTORC1 hyperactivity, promoting impaired mitochondrial dynamics and resulting in damaged, fragmented mitochondria. The accumulation of damaged mitochondria impairs oxidative phosphorylation, reducing ATP, increasing reliance on the lactate shuttle, and increasing ROS and DNA damage. These changes resulting from mitochondrial damage then produce an environment that activates pathways that suppress mTORC1, potentially explaining why mTORC1 is observed to be downregulated in more chronic, established schizophrenia, despite the proposed earlier hyperactivation. Chronic, multiple-episode schizophrenia then has impaired oxidative phosphorylation machinery, while also having downregulated glucose transport into the brain, reducing both its ability for oxidative and glycolytic metabolism, forcing a reliance on lactate as its primary fuel source. However, once again, this mechanism remains highly theoretical and requires substantial longitudinal research to be verified.

#### 4.3.6. PI3K/AKT and mTOR’s Role in Synaptic and Structural Abnormalities

Previous sections suggest a theoretical mechanism by which insulin resistance and reduced insulin signalling could disrupt neurotransmitter function through the PI3K/AKT pathway. However, evidence also demonstrates that the PI3K/AKT signalling pathway regulates neural progenitor proliferation, neuronal survival, migration, neurite outgrowth, synaptogenesis, and myelination, while mTOR acts as a major downstream effector coordinating protein synthesis and cellular growth required for these processes [257,258].

PI3K/AKT phosphorylation of BAD inhibits its pro-apoptotic activity, promoting cellular survival [259]. PI3K/AKT inhibition of FOXO prevents the activation of FASL and BIM, two pro-apoptotic proteins [50]. However, expression of FOXO is decreased or dysregulated in schizophrenia patients, which we would not expect given reduced AKT expression [260]. In insulin-resistant states, ERK signalling can remain active despite AKT suppression, and ERK has also been shown to downregulate FOXO [261,262,263,264]. Similarly, PI3K/AKT phosphorylation of Mdm2 prevents the activation of genes that induce cell-cycle arrest or trigger apoptosis [265]. Ubiquitin-related proteins, such as Mdm2, are decreased in schizophrenia and are associated with neuronal survival, morphology, and memory [266]. Given PI3K/AKT’s role in regulating apoptosis, and evidence from schizophrenia consistently finding decreased expression and activity, we may expect to see an increase in neural loss. However, schizophrenia is not considered a classical neurodegenerative disorder; it does not have widespread neuronal destruction and instead shows reduced dendritic arborization, reduced neuropil, synaptic abnormalities, and cortical thinning [267,268].

There may be several mechanisms that prevent apoptosis. The intrinsic, or mitochondrial, apoptosis pathway is activated due to intracellular stress, which causes a signalling cascade to mitochondrial outer membrane permeabilisation (MOMP), leading to the release of cytochrome c and consequently the formation of the apoptosome [269]. BAX/BAK are the key pro-apoptotic effectors of intrinsic apoptosis, forming pores in the mitochondrial outer membrane and being regulated by anti-apoptotic proteins such as Bcl-2 and Bcl-xL. Although reduced PI3K/AKT signalling would be expected to lower the apoptotic threshold, commitment to this intrinsic apoptosis is thought to occur at the level of BAX/BAK-mediated MOMP, which is regarded as the apoptotic “point of no return” [270,271]. Similarly, some reviews have suggested that apoptosis shares similar molecular features to synaptic pruning, and can be localised to surrounding neurites, without inducing immediate neuronal death or involving the neuron’s cell body [272]. Evidence in schizophrenia may support this, with local apoptosis occurring in schizophrenia with an absence of neuronal cell loss and reductions in pyramidal neuron size [273]. Furthermore, schizophrenia has been associated with increased BAX/Bcl-2 ratios and decreased Bcl-2 levels in the postmortem tissue of different brain regions, without increased caspase 3 levels, indicating that increased apoptosis signalling does not progress to execution-phase apoptosis [272,274]. Together, these findings may suggest that AKT suppression may not reach a high enough threshold for neural apoptosis; instead, apoptotic signalling in schizophrenia may be spatially restricted to dendrites and synapses, resulting in local pruning. However, these processes are not well understood, and a full analysis is beyond the scope of this review.

Another important structural mechanism associated with PI3K/AKT may involve microtubule function. PI3K/AKT-mediated inhibition of GSK3 reduces Tau phosphorylation, which, when this signalling cascade functions normally, helps stabilise microtubules [275]. Stabilisation of microtubules through Tau plays a role in maintaining neuronal structure, transporting cellular cargo, and is essential in neuronal migration, differentiation, and maturation [276]. Low circulating levels of tau have been reported in both adult and adolescent schizophrenia patients compared to healthy controls, independent of antipsychotic use or dosage [277,278]. GSK3 also regulates several other microtubule-associated proteins, including MAP, CRMP, and APC, which have also been shown to be dysregulated in schizophrenia [279,280,281,282,283]. These microtubule-associated proteins play a crucial role in the formation and maintenance of neuronal architecture [284]. Under normal physiological conditions, AKT-mediated inhibition of GSK3 promotes microtubule stabilisation and facilitates neurite outgrowth, processes that are essential for neuronal polarisation, axonal extension, and the establishment of dendritic arbours during development [276,284]. Similarly, the growth and branching of dendrites depend on tightly regulated microtubule remodelling, with cycles of polymerisation, stabilisation, and reorganisation that support dendritic elongation and arborization [285]. mTOR activation also plays a significant role by promoting the synthesis of proteins required for dendritic growth, structural plasticity, and neuronal maturation through the regulation of protein translation machinery, including S6K and 4E-BP [286]. These findings suggest that PI3K/AKT/mTOR signalling contributes to dendritic development through coordinated regulation of both cytoskeleton organisation and protein synthesis.

PI3K/AKT and mTOR have also been shown to regulate oligodendrocyte development and CNS myelination. Experimental mouse models have demonstrated that AKT regulates myelination largely through activation of mTOR signalling, with AKT activation increasing myelin thickness and leading to hypermyelination in the CNS during development [287]. Subsequent work showed that mTOR activity is essential for oligodendrocyte differentiation, as pharmacological inhibition of mTOR stopped oligodendrocyte precursor cells at the late progenitor stage and prevented their maturation into myelinating oligodendrocytes [288]. Similarly, oligodendrocyte-specific deletion of mTOR reduced the number of mature oligodendrocytes, delayed the onset of myelination, and resulted in persistent hypomyelination in vivo, suggesting that mTOR regulates not only differentiation but also the initiation and extent of myelin sheath formation [289]. Further research examining this mechanism used TSC1 knockout mice, which causes overactivation of mTORC1, leading to hypomyelination by downregulating AKT signalling and lipogenic pathways, suggesting that mTOR function needs to be tightly balanced for normal myelination processes [290]. Schizophrenia has been associated with dysregulation of key oligodendrocyte and myelination genes, including transcription factors that regulate these genes [291]. Similarly, evidence of myelination and oligodendrocyte abnormalities has been consistently observed across multiple brain networks in postmortem and neuroimaging studies, with several suggesting that this widespread reduction in white matter may impair communication between brain regions and create cognitive deficits [292,293,294].

Taken together, converging evidence of PI3K/AKT dysregulation due to insulin resistance and reduced insulin signalling could lead to the widespread white matter abnormalities, abnormal pruning, and dendritic developmental abnormalities that have been associated with schizophrenia.

## 5. Convergence with Contemporary Schizophrenia Theory

The above sections provide a theoretical mechanistic framework detailing how genetic vulnerabilities involving insulin resistance and reduced insulin signalling associated with schizophrenia may lead to dysregulation of the PI3K/AKT and mTOR signalling pathways, which affect multiple metabolic cascades, causing neuroinflammation, mitochondrial damage, and ultimately neurological dysfunction. However, given that metabolism is only a recently developing field in schizophrenia research, there is not enough evidence to establish the directionality of the described mechanistic pathway or to suggest that early insulin resistance and hypoxic injury are causing the associated downstream abnormalities. It may also be that mitochondrial genetic risk factors produce the outcomes associated with the mechanism described above in reverse.

An early review by Flippo and Strack [242] on mitochondrial dysfunction in schizophrenia highlights that several mtDNA SNPs, along with other SNPs involved in mitochondrial respiration, are strongly associated with the development of schizophrenia. Genetic abnormalities in mitochondrial respiration and biogenesis may indicate that certain subpopulations begin at the final metabolic shift detailed in the above mechanism and develop insulin resistance and dysregulation of the PI3K/AKT/mTOR pathways through a dysfunctional mitochondria-mediated feedback mechanism. The directionality of insulin resistance and mitochondrial dysfunction in diabetes research remains highly debated, suggesting a complex bidirectional relationship [295]. For example, Petersen et al. [296], whose work combines human metabolic studies with mechanistic models of lipid-induced insulin resistance, suggest that inherited mitochondrial defects may precede the development of insulin resistance. They propose that impaired mitochondrial oxidative capacity reduces fatty acid oxidation, leading to the accumulation of lipid intermediates, which activate stress kinases and induce insulin resistance at the level of IRS and AKT [297].

Regardless of the ongoing discussion surrounding the directionality of IR and mitochondrial dysfunction, current evidence for their role in PI3K/AKT and mTOR dysfunction is relatively clear and may suggest that there are two distinct metabolic subtypes of schizophrenia which converge on dysregulation of this pathway. Similarly, it is also plausible that other non-metabolic genetic risk factors are the primary drivers of schizophrenia development, which are only exacerbated by insulin resistance and mitochondrial dysfunction. Nonetheless, this metabolic and cell-signalling framework may provide a unifying model, explaining several underlying mechanistic steps that converge with other contemporary theories of schizophrenia.

### 5.1. Prenatal Developmental Models

There are several known prenatal factors associated with increased risk for schizophrenia development, including prenatal maternal infection, immune activation, obstetric complications, malnutrition, maternal stress, and some small seasonal effects [298]. These risk factors are often clustered under the neurodevelopmental hypothesis of schizophrenia and are suggested to cause abnormalities in brain structure and function during gestation [299]. Interestingly, maternal diabetes has been associated with a high-risk factor for schizophrenia development, having 7.76 times higher odds of development than healthy groups [300]. This association between maternal diabetes and schizophrenia risk has been suggested to be due to hypoxia, ROS, iron deficiencies, and inflammation, impairing the normal and healthy development of the foetus [301]. However, around 7% of women have diabetes during pregnancy, and only a small portion of children go on to develop schizophrenia, suggesting that gestational complications associated with diabetes do not predetermine schizophrenia, and simply increase the risk [302]. Here, the mechanisms and genetic risk factors described above may create a prenatal and placental environment characterised by ROS, hypoxia, nutrient deficiency, and inflammation, which causes prenatal developmental damage and creates further vulnerabilities that perpetuate dysregulation into infancy and early adolescence.

Interestingly, there is growing evidence that foetal hypoxia drives a Warburg-like effect, downregulating oxidative metabolism and shifting to glycolysis [303]. An increase in ROS production and a decrease in antioxidant defences are also associated with foetal hypoxia, which has been suggested to result from mitochondrial dysfunction [304,305,306]. Similarly, miR-137, mTOR and AKT have also been implicated in several prenatal developmental abnormalities and have been suggested to dysregulate placental and neural development in schizophrenia [307]. A prenatal foetus is also more vulnerable to the mechanical pathways detailed above, as it has immature antioxidant defences and underdeveloped mitochondrial quality-control mechanisms (mitophagy, fusion, and fission) [308,309,310,311].

Animal models of hypoxia in gestation have also been associated with sustained suppression of oxidative phosphorylation, reduced ATP production, reduced mitochondrial respiration, elevated basal ROS generation, persistent oxidative stress, reduced SOD/antioxidative defences, reduced PGC-1α signalling, and persistent metabolic reprogramming after birth [312,313,314,315,316,317]. Interestingly, most of the evidence suggests males are at a higher risk for damage that persists after birth, which may suggest a risk mechanism for the slightly higher rates of schizophrenia in males than females [318]. However, due to obvious ethical concerns, most evidence has been collected in cardiovascular cells or from animal models, and there is a significant research gap regarding these mechanisms in human models and brain tissue. Nonetheless, current evidence suggests that prenatal hypoxia may impair several components of the mechanism described above; of particular interest are reductions in PGC-1α and antioxidant defence.

As mentioned earlier, PGC-1α and SIRT3 are responsible for mitochondrial health and antioxidant defence in neurons, astrocytes, and microglia, preventing the accumulation of ROS, allowing normal oxidative metabolism to occur, and, importantly, switching microglia back to their anti-inflammatory state [211,223,224]. If these mechanisms are damaged prenatally, it may indicate a mechanism by which neurons, astrocytes, and microglia become locked out of oxidative phosphorylation, as mitochondria and mitochondrial repair mechanisms have been damaged, preventing a reversal to oxidative metabolism, leading to further metabolic damage, insulin resistance, mitochondrial dysfunction, and changes in redox signalling.

These early mitochondrial insults and the switch away from oxidative phosphorylation may drive dysregulation of the PI3K/AKT and mTOR cell-signalling cascades at key developmental time points in the adolescent brain. As detailed in earlier sections, experimental evidence demonstrates that the PI3K/AKT signalling pathway regulates neural progenitor proliferation, neuronal survival, migration, neurite outgrowth, synaptogenesis, and myelination, while mTOR acts as a major downstream effector coordinating protein synthesis and cellular growth required for these processes [257,258]. PI3K/AKT and mTOR carry out this through their responsiveness to growth factors, and their activation contributes to the expansion and maintenance of neural progenitor populations, supports neural survival by suppressing apoptotic signalling, and regulates cytoskeletal remodelling required for neuronal migration and maturation. In addition, the pathway influences dendritic arborization, synaptic protein synthesis, and oligodendrocyte development, suggesting that disrupting this pathway during development could have widespread consequences for cortical circuit formation and connectivity throughout development [258].

These suggested mechanisms also align with emerging neurodevelopmental theories that suggest aerobic glycolysis dysfunction as a compensatory mechanism during development but ultimately leads to maladaptive outcomes in adulthood [319]. Glucose consumption and aerobic glycolysis remain high across childhood and slowly decline in adolescence, reaching the mature oxidative metabolic pattern in young adulthood (approximately 20 years old) [320]. Alongside these metabolic changes, adolescence and young adulthood represent a prolonged period of neural maturation, characterised by synaptic pruning, changes in neurotransmitter systems, and continued refinement of prefrontal circuits [321]. This shift from aerobic glycolysis to oxidative phosphorylation aligns with the proposed mechanism, where this switch may expose underlying antioxidant defence and mitochondrial vulnerabilities, resulting in insulin resistance and the metabolic changes described above, during a particularly important neurodevelopmental window. Evidence from Alzheimer’s research may support this theory, with one study suggesting developing neurons in glycolysis are unimpaired by mitophagy deficiency [322]. However, once they switch towards oxidative phosphorylation, mitophagy becomes severely impaired.

### 5.2. Synaptic, Connectivity, and Neurotransmitter Models

Beyond its potential role in metabolism, inflammation, and neurodevelopment, this framework may also intersect with contemporary neurotransmitter, synaptic dysfunction, and dysconnectivity theories of schizophrenia. Growing evidence suggests that these models are not independent explanations of disease pathophysiology but rather describe different levels of dysfunction that emerge across development [10]. The evidence presented here suggests that impaired insulin signalling and metabolic dysfunction converge on the PI3K/AKT/mTOR signalling network. While current evidence does not establish PI3K/AKT/mTOR dysfunction as a primary cause of schizophrenia, the pathway occupies a strategic regulatory position that converges with contemporary theories of schizophrenia.

Reduced PI3K/AKT signalling provides a mechanistic explanation for neurotransmitter abnormalities by impairing GABAergic inhibition and altering dopaminergic regulation within mesolimbic and mesostriatal circuits, thereby linking directly to both the glutamate/GABA and dopamine hypotheses. At the synaptic level, PI3K/AKT/mTOR signalling regulates dendritic growth, synaptic protein synthesis, synaptic plasticity, receptor trafficking, and neurotrophic function; consequently, chronic dysregulation of this pathway may contribute to synaptic loss, impaired plasticity, and aberrant pruning proposed by synaptic models of schizophrenia. Similarly, the downstream targets of PI3K/AKT and mTOR play essential roles in neuronal migration, axonal growth, oligodendrocyte maturation, and myelination, providing a biologically plausible link to neurodevelopmental and dysconnectivity theories of schizophrenia. Consequently, this theoretical mechanistic framework does not directly challenge or replace contemporary frameworks of schizophrenia development and instead provides a unifying mechanism that explains several of the abnormalities associated with the pathophysiology of the disorder.

## 6. Overlap with Mood Disorders

It is also worth noting that depressive disorders and bipolar disorders are frequently found to have abnormalities in the above framework, including insulin resistance, mitochondrial dysfunction, and dysregulation of the PI3K/AKT and mTOR signalling pathways [323,324,325,326,327]. Depression and bipolar disorder have estimated insulin resistance prevalence rates of 30–45% and 24–41%, respectively [328,329]. Given the significant overlap between schizophrenia, bipolar and depressive disorders, examining where they diverge mechanistically in the framework and across development may provide insights into their aetiology. Table 1 indicates significant overlap among insulin resistance, PI3K/AKT, mTOR and mitochondrial dysfunction mechanisms between schizophrenia, bipolar disorder, and depression.

Unlike schizophrenia, depressive disorder is generally conceptualised as a disorder of impaired adult neuroplasticity and stress adaptation, with the median age of onset occurring at approximately 25 years [366]. Although early-life adversity and developmental factors contribute to vulnerability, the core pathological processes are thought to involve dysregulation of neural circuits, stress-response systems, inflammation, cellular metabolism, and synaptic plasticity rather than primary abnormalities in early brain development [366]. Perhaps the most prevalent risk factor in depression compared to schizophrenia is stressful life events. Depression is strongly associated with recent or chronic stress, and substantial evidence implicates hyperactivity of the HPA axis (hypothalamic–pituitary–adrenal) as a key biological consequence linking stress to depressive pathophysiology [366]. Similarly, chronic stress is increasingly recognised as an important contributor to the development of insulin resistance. Recent evidence suggests that sustained stress disrupts glucose homeostasis through multiple interconnected mechanisms, including prolonged activation of the HPA axis, glucocorticoid excess, sympathetic nervous system activation, chronic inflammation, and altered adipokine signalling, collectively impairing insulin signalling and promoting insulin resistance [367]. This stress response and the resulting insulin resistance may be among the risk factors contributing to the dysregulation of the PI3K/AKT and mTOR pathways described above, leading to dendritic spine and white matter loss, alongside neurotransmitter dysfunction, inflammation, and metabolic abnormalities.

Although schizophrenia and depression share abnormalities within the prefrontal cortex and hippocampus, the pattern and interpretation of these structural changes differ considerably. Schizophrenia is characterised by widespread abnormalities involving the dorsolateral prefrontal cortex, superior temporal cortex, hippocampus, thalamus, anterior cingulate cortex, insula, and frontotemporal white matter tracts, with the greatest alteration occurring in association cortices that undergo prolonged postnatal development, synaptic refinement, dendritic maturation, and myelination throughout adolescence and early adulthood [368,369]. In contrast, structural abnormalities in depression are more regionally restricted, primarily involving fronto-limbic circuits, including the medial prefrontal cortex, anterior cingulate cortex, hippocampus, amygdala, and ventral striatum, which regulate mood, cognition, and reward processing [370]. Rather than reflecting abnormal brain development, these changes are generally interpreted as consequences of chronic stress, characterised by dendritic retraction, synaptic loss, impaired neuroplasticity, and reduced neurogenesis within stress-sensitive brain regions [371,372]. Together, this evidence may suggest that IR, mitochondrial dysfunction and the PI3K/AKT/mTOR pathway contribute to schizophrenia during key developmental periods, leading to more severe abnormalities in brain structures, whereas this pathway contributes to depression after development and impacts stress-sensitive regions.

Table 1 suggests that bipolar disorder may be characterised by dysregulation of this pathway rather than a homogeneous increase or reduction. Compared to schizophrenia and depression, bipolar disorder is uniquely characterised by prominent disturbances in circadian rhythms and sleep–wake regulation, which are among the strongest predictors of mood episode onset and recurrence [373]. Consequently, contemporary models increasingly conceptualise bipolar disorder as a disorder of chronobiology, where instability of circadian timing contributes to transitions between manic and depressive states [374]. Interestingly, circadian regulation is tightly integrated with cellular metabolism rather than functioning as an independent biological system. Circadian clock genes regulate daily oscillations in insulin secretion, insulin sensitivity, glucose metabolism, mitochondrial respiration, oxidative phosphorylation, protein synthesis, and autophagy, while nutrient-sensitive signalling pathways, including PI3K/AKT, mTOR, AMPK, and GSK3β, reciprocally regulate the molecular clock through effects on CLOCK, BMAL1, PER, and CRY proteins [375,376,377]. Circadian rhythm dysfunction and sleep deprivation have also been associated with altered glucose metabolism, insulin resistance, hyperglycaemia, metabolic syndrome, and type 2 diabetes risk [378,379,380,381,382,383]. Circadian dysregulation may therefore represent an upstream perturbation that converges on the insulin, PI3K/AKT, and mTOR pathways, leading to compromised neural energy homeostasis and synaptic plasticity. Early evidence may support this theory, as some animal model studies show that elevated mTOR activity is associated with manic states, and human studies show that reduced mTOR activity is associated with depressive states [337].

These disorders share several additional environmental risk factors. Childhood adversity, systemic inflammation, obesity, unhealthy dietary patterns, physical inactivity, socioeconomic deprivation, and chronic psychosocial stress have all been consistently associated with increased risk of severe psychiatric illness, although their relative contribution differs across diagnostic groups [299,366,384,385]. Importantly, many of these same exposures are also independently associated with the development of insulin resistance. Childhood trauma promotes long-term HPA-axis dysregulation and chronic low-grade inflammation; obesity and poor diet directly impair insulin signalling; physical inactivity reduces insulin sensitivity and mitochondrial function; while inflammatory states promote serine phosphorylation of IRS proteins, collectively disrupting PI3K/AKT signalling [180,367,386,387]. Drug use, particularly cannabis, is a known risk factor for schizophrenia and bipolar disorder, and to a lesser extent depression [388,389]. Interestingly, cannabis use has also been associated with reduced mitochondrial respiration, dysregulation of mitochondrial membranes, and increased ROS production, potentially suggesting another risk factor interacting with the suggested mechanism [390,391].

Overall, chronic stress and HPA-axis hyperactivity are particularly characteristic of depression; circadian disruptions are most strongly associated with bipolar disorder; and prenatal damage is considered a major environmental contributor to schizophrenia, which, alongside other environmental risk factors, converges on insulin resistance, mitochondrial dysfunction, PI3K/AKT, and mTOR function. Ultimately, current evidence may suggest that the PI3K/AKT, mTOR, insulin resistance, and mitochondrial dysfunction pathways may be transdiagnostic and underpin severe mental illness, with disorders diverging through developmental timing and environmental risk factors. However, more longitudinal research is needed to examine these pathways across the development of these disorders.

## 7. The Role of Diet

Individuals suffering from schizophrenia are at increased risk for a poor diet, often characterised by increased sugar intake, cholesterol, and saturated fat, while avoiding healthier options [392]. High-fat and high-sugar diets have been shown to reduce brain-derived neurotrophic factors, which have been associated with schizophrenia [392]. Ecological studies have shown that increased sugar intake is associated with poor two-year outcomes in schizophrenia [393].

The ketogenic diet has historically been used in treating epilepsy, showing that up to 50% of participants had a reduction in seizure severity and frequency [394]. It is also well known that interventions that are effective for epilepsy also show significant positive results for psychiatric disorders. Recently, the ketogenic diet has been gaining traction in the literature as an effective treatment intervention for schizophrenia and bipolar disorder. Several case studies have generated early positive results. For example, two patients diagnosed with schizophrenia, aged 82 and 39 years old, were put on ketogenic diets for unrelated health reasons [394]. Both individuals had complete remission of their psychotic symptoms in one to three months and are now fully independent while remaining on the ketogenic diet. Another study by Palmer [395] using the ketogenic diet to treat schizoaffective disorder showed significant decreases in PANSS (scale for measuring schizophrenia symptom severity). A 70-year-old female with chronic schizophrenia had her hallucinations and delusions disappear after 8 days on the diet, and remained in remission with no recurrence over 12 months [396]. However, this was a weak case report because ketosis was assumed rather than measured directly. Two opposite sex twins with schizophrenia were also put on the ketogenic diet and saw moderate PANSS score reductions [397]. However, the study period on ketosis was short (2 weeks), and diet adherence was poor. Two women with chronic schizoaffective disorder (17 and 32 years old) experienced full remission of symptoms and reduced their medication intake over 24-week and 52-week periods, respectively [398]. Converging evidence from mouse models of schizophrenia has generated similar positive results from the ketogenic diet or ketone body treatment [399,400].

A retrospective analysis by Danan et al. [401] examined the effect of the ketogenic diet on 28 participants with either major depressive disorder, bipolar disorder, or schizoaffective disorder. For the 18 individuals with depression and bipolar disorder, mean depression ratings on the Hamilton Depression Rating Scale and Montgomery–Åsberg Depression Rating Scale significantly improved (from 25.4 to 7.7 and from 29.6 to 10.1, respectively). For the 10 individuals with schizoaffective disorder, the mean PANSS scores improved from 91.4 to 49.3. A more recent pilot trial by Sethi et al. [402] examined the effects of the ketogenic diet on bipolar disorder and schizophrenia. Twenty-three participants with schizophrenia or bipolar disorder and existing metabolic disorders were put on the ketogenic diet for four months. Results showed remission of metabolic disorder, with none of the participants meeting the criteria for metabolic syndrome by the end of the study. Participants also experienced significant body recomposition, and metabolic markers showed improvements, including HbA1c reduction by 3.6% and HOMA-IR (a measure of insulin resistance) by 27% (indicating improved glycaemic control and insulin sensitivity). Participants with schizophrenia had a 32% reduction in brief psychiatric rating scores. Overall, clinical global impression severity (a scale assessing the severity of mental health illnesses) improved by 31% on average, and life satisfaction and sleep quality improved by 17% and 19%, respectively. Qualitatively, participants reported significant decreases in anxiety and mood stabilisation, and some believed that remission was possible. More recent clinical trials and meta-analyses for depression show similar results, with the ketogenic diet producing antidepressant effects compared to other well-matched diets [403,404]. However, most research has been observational with weak data, and further trials are required to examine the diet’s true effects; thus, these initial results should be treated with caution. Nonetheless, early evidence suggests that the ketogenic diet may be an effective intervention for depressive and schizophrenia-spectrum disorders. The question then arises: how does the ketogenic diet impact the cell-signalling and metabolic mechanisms detailed above?

Nutrition and metabolism are deeply linked, synergistic, and homeostatic [405,406]. For example, the ketogenic diet is a high-fat, low-carbohydrate diet that shifts metabolism and provides the brain with ketone bodies such as β-hydroxybutyrate (BHB), acetoacetate (AcAc), and acetone as an alternate energy source to glucose [407]. In the presence of low insulin concentrations, ketogenesis converts acetyl-CoA from fatty acid oxidation into BHB and AcAc, whereas spontaneous AcAc decarboxylation produces acetone at a significantly reduced rate compared to BHB and AcAc production [408]. Ketogenesis mainly occurs in the liver, but BHB and AcAc can cross the BBB via monocarboxylate transporters (MCTs), which are upregulated in the BBB due to elevated ketone levels [409]. Ketogenesis also occurs in astrocytes and microglia, andthe brain is able to derive roughly two-thirds of its metabolic requirements from ketone oxidation [410,411,412]. Ketones supply acetyl-CoA directly to the TCA cycle, bypassing glycolysis and maintaining oxidative ATP production independently of glycolytic flux, thereby reducing mitochondrial energy production’s dependence on insulin-mediated glucose uptake [413,414].

This switch to ketones as a primary energy source causes a range of metabolic changes that are often analogous to fasting states, including lower glucose, lower insulin levels, improved insulin sensitivity, reduced lipogenesis, reduced triglyceride accumulation, and a significant reduction in the nutrient overload associated with insulin resistance [415,416,417,418]. Given the reduction in insulin resistance and nutrient overload associated with the ketogenic diet, it is perhaps unsurprising that it has been associated with changes in the PI3K/AKT and mTOR pathways. A recent systematic review by Matawali, Yeap, Sulaiman and Tan [408] examined the effects of ketone bodies and ketogenesis on the PI3K/AKT/mTOR pathway. Across most of the included literature, ketone bodies (particularly BHB) and ketogenic states (ketogenic diet, fasting, or exposure to exogenous ketone bodies) tended to be associated with reductions in phosphorylation of AKT, mTOR, and mTOR’s downstream targets S6K and 4E-BP1, across cellular lines, animal studies, and a limited number of human studies [408]. Taken together, this evidence suggests that switching to the ketogenic diet removes the environmental glucose factor that interacts with genetic risk, thereby reducing insulin resistance, bypassing glycolysis, maintaining oxidative metabolism, and preventing the mTORC1 dysfunction mentioned above.

Interestingly, the ketogenic diet has also been shown to impact mitochondrial function, antioxidant defence, reduce ROS-mediated damage, and suppress inflammatory and oxidative signalling [414]. These mitochondrial changes may suggest that the ketogenic diet not only bypasses initial vulnerabilities but also mitigates their downstream effects, as indicated by the final metabolic shift. Evidence from mouse models suggests that ketogenic metabolism increases NADH oxidation and respiration capacity, ultimately reducing electron leakage from the ETC and reducing ROS generation [419]. Similar mouse model studies suggest that the ketone body BHB increased mitochondrial membrane potential, increased the NAD+/NADH ratio, and enhanced FOXO and PGC-1α in a SIRT2-dependent manner, thereby increasing autophagy, mitophagy, and mitochondrial biogenesis [420]. An increased NAD+/NADH ratio is known to increase activation of the SIRT pathways, including SIRT3 [412]. As mentioned earlier, SIRT3 is an NAD-dependent deacetylase and is responsible for mitochondrial health and antioxidant defence in neurons and astrocytes [221,421]. SIRT3 deacetylates and activates manganese superoxide dismutase (SOD2) and several proteins in complex I and II of the ETC, which allows SOD2 to convert harmful superoxide radicals into hydrogen peroxide and oxygen, and deacetylation of ETC complexes increases TCA metabolic flux [422,423]. Further evidence from mouse models also suggests that the ketogenic diet upregulates mitochondrial uncoupling protein (UCP) activity, leading to increased UCP-mediated proton conductance, reduced ROS production and increased mitochondrial respiration [424].

Perhaps the most comprehensive study examining the effects of ketones on mitochondria was conducted by Hasan-Olive et al. [425], who examined mouse models with a mutated mitochondrial DNA repair enzyme, cultured rat hippocampal neurons, and human fibroblasts under induced oxidative stress. Their findings showed that the ketogenic diet, or BHB exposure, improved markers of mitochondrial biogenesis in neurons under oxidative stress by increasing PGC-1α, SIRT3, and UCP2, raising oxygen consumption rates and the NAD+/NADH ratio. Furthermore, UCP2 levels were elevated in hippocampal neurons of keto-diet mice, and the ketone receptor GPR109a (also known as HCAR2) was upregulated, suggesting ketone-mediated signalling. BHB ketone exposure has also been suggested to modulate inflammatory pathways [426]. BHB has been shown to inhibit activation of the NF-κB pathway in an HCAR2-mediated manner, leading to decreased pro-inflammatory cytokines in rat microglial cells [427,428]. Similarly, in mouse models and human monocytes, BHB has been shown to reduce the NLRP3 inflammasome, leading to reduced IL-1β and IL-18 (interleukin) production in immune cells [429]. Taken together, the current evidence suggests that the ketogenic diet may improve mitochondrial function by increasing the NAD+/NADH ratio, thereby activating the PGC-1α/SIRT3/UCP2 signalling cascade, which then reduces ROS production, clears already produced ROS, and increases mitochondrial respiration. Similarly, this reduction in ROS may attenuate pro-inflammatory signalling, while the ketone body BHB directly inhibits key pro-inflammatory pathways, including NF-κB and the NLRP3 inflammasome.

Taken together, evidence surrounding the ketogenic diet suggests that it interacts with our framework of schizophrenia and mood disorders at several different points. First, the initial diet change and switch to ketogenesis bypass glycolysis, supplying the TCA cycle with acetyl-CoA directly, allowing oxidative ATP production to continue independently of insulin-mediated glucose uptake. The flow-on effects of this switch to ketosis (improved insulin sensitivity, reduced nutrient overload) during the development of schizophrenia or in bipolar disorder may attenuate the dysregulated mTOR pathways and reduce insulin resistance, allowing PI3K/AKT to return to normal functioning levels, and attenuating the abnormal downstream impacts associated with their hyperactivation/suppression.

## 8. Future Directions

Given that the framework is largely theoretical, the central argument is based on data synthesised across multiple fields. Currently, the theory is a system-level hypothesis with limited causal validation from longitudinal or interventional studies. However, due to ethical considerations, studies on developmental timing, intracellular signalling in neurons, and full developmental trajectories will likely remain largely inferential and will require synthesised analyses of human, animal, and cellular evidence rather than direct proof. Nonetheless, several aspects of the framework are supported by limited or weak evidence, and future research could address these limitations.

First, there are no direct studies examining hyperglycaemia-induced ER stress and SERCA pump activity, which lead to Ca^2+^ dysregulation in human neurons. Second, mechanisms of HIF-1α dysregulation in schizophrenia and bipolar disorder are speculative, with very little data in brain tissues and inconsistent findings across other tissues and animal models. Third, the link between glycolysis (mTOR/HIF-1α) and M1/M2 microglial states is well supported in immunology but has not been directly examined in schizophrenia and bipolar disorder under hyperglycaemic conditions. Finally, evidence for mTOR-mediated impairment of mitophagy and mitochondrial fragmentation is inferred from genetic, animal, or non-neuronal studies. Future research could begin addressing these limitations using cell culture methods.

Another significant limitation concerns the microbiota–gut–brain axis. Recently, psychiatric research has given significant attention to the gut microbiota, as current evidence indicates that these bacteria act as ‘metabolic machinery’ that can influence several mechanisms considered core features of mental disorders [430]. These symbiotic bacteria influence the CNS through the microbiota–gut–brain axis and influence neural, hormonal, and immunological pathways [431,432,433]. A recent review by McGuinness, Davis, Dawson, Loughman, Collier, O’Hely, Simpson, Green, Marx, Hair, Guest, Mohebbi, Berk, Stupart, Watters and Jacka [430] highlighted the significance of gut microbiota, showing that schizophrenia, bipolar disorder, and major depressive disorder consistently show differences in β diversity between healthy and control participants. Furthermore, their analysis identified lower levels of bacterial genera that produce short-chain fatty acids, higher levels of lactic-acid-producing bacteria, and higher levels of bacteria associated with GABA and glutamate metabolism [430]. Interestingly, findings from gut–microbiota studies in type 2 diabetes follow a similar profile found in schizophrenia, bipolar, and major depressive disorders, showing a reduction in short-chain fatty acid producers, altered β diversity, and increased lactic-acid-producing bacteria [434]. However, GABA and glutamate metabolism have not been reported. Nonetheless, these similarities between gut-microbiome changes may suggest a significant link with our mechanistic framework; however, the evidence is unclear on whether the gut–brain axis is associated, causal, or confounded in these disorders, and a full, detailed analysis is beyond the scope of this review [435,436].

Despite the theories’ current weaknesses, this expanded framework suggests several areas for future research and has several clinical implications. Current psychiatric medications for schizophrenia and bipolar disorder target dopamine D2 receptor antagonism, reducing mesolimbic dopamine, or use 5-HT2A serotonin antagonism/partial agonism to modulate dopamine in cortex/striatal circuits, resulting in a reduction in positive symptoms [437,438]. The most effective antipsychotics typically have a moderate effect size (~0.5) compared to placebo treatments for schizophrenia, and manic bipolar disorder typically has a moderate to large effect size (~0.5–0.7) [439,440]. However, these drugs often only target positive symptoms (mania, psychosis, and severe mood swings), while having a limited effect on negative symptoms (anergia, avolition, and cognitive deficits) [441]. Furthermore, they can increase negative symptoms, and commonly produce adverse side effects such as constipation, akathisia (inner restlessness), sexual dysfunction, acute dystonias (painful muscle contractions), weight gain, tardive dyskinesia (involuntary, tic-like movements), and myocarditis (heart inflammation) [442]. However, perhaps the most important negative outcome associated with antipsychotics (and to a lesser extent, mood stabilisers) are the strong negative metabolic outcomes associated with their use. Antipsychotic use leads to weight gain (42–62%) and obesity, lipid abnormalities, insulin resistance, hyperglycaemia, an increased risk of diabetes, dyslipidaemia, and hypertension [443,444,445,446].

One might suggest that these metabolic outcomes associated with antipsychotic use are confounded by the underlying genetic metabolic risk factors. However, several healthy cell cultures, rat models, and acute human studies suggest that these antipsychotic drugs directly cause metabolic dysfunction. For example, healthy male rat models treated with olanzapine and haloperidol (dopamine antagonists, reducing dopamine activity and psychosis symptoms) show significant weight gain, increased plasma insulin, and higher HOMA-IR (insulin resistance) after just 8 weeks of treatment [447,448]. Interestingly, these effects were attributed to decreased IRS1/2 activity and increased mTOR activity. Another recent rat model and cell culture study of olanzapine by S. Li et al. [449] showed similar results, with increased glycolysis and ATP production; however, the PI3K/AKT pathway was inhibited, potentially leading to increased lipogenesis (the conversion of glucose into fats). IR persists with chronic use and leads to increased production of inflammatory cytokines (IL-1β, IL-6, IL-8, and TNF-α), further exacerbating IR [450]. It must be noted that these studies have been conducted on peripheral cells, and one could suggest that this does not impair brain function. However, even if these drug effects do not directly affect brain function, they may indirectly impair it, as brain IR likely arises secondarily to peripheral factors that impair insulin transport across the BBB [451,452,453].

Evidence also suggests that antipsychotics impair mitochondrial function in the schizophrenia brain, particularly complex I, leading to impaired oxidative phosphorylation and reduced ATP production [454]. However, it is difficult to determine whether these mitochondrial changes result from normal disease progression or drug use, and clinical trials are still underway to understand these mitochondrial effects [455]. Finally, antipsychotics also produce hyperphagia (insatiable hunger), increased food intake, loss of control over eating, and changes in food-related reward and cravings by altering hypothalamic signalling and leptin/ghrelin pathways [456,457]. This food-behaviour-based antipsychotic effect could be a further confound, producing metabolic changes through eating behaviours rather than direct drug effects. Despite these uncertainties, antipsychotic treatment worsening or having little effect on negative symptoms while only improving positive symptoms may be due to treating downstream neurotransmitter effects, while worsening the underlying cellular signalling and metabolic mechanisms.

Metformin has recently emerged as a potential treatment mechanism in treatment-resistant bipolar disorder, with a randomised, masked, and placebo-controlled clinical trial by Calkin et al. [458] showing significant reductions in insulin resistance (no longer meeting criteria for IR) and several depression/bipolar scales. Metformin is typically prescribed for type 2 diabetes and reduces glucose production, increases glucose utilisation, and increases GLP-1 production [459]. Metformin also inhibits the mitochondrial respiratory chain (particularly at complex I), reducing ATP and increasing AMP, which activates AMPK and ultimately decreases the expression of gluconeogenic enzymes [460]. Interestingly, metformin is only a mild inhibitor of complex I and has only a small net reduction in oxygen consumption [461,462,463]. Thus, metformin’s mechanism of action fits nicely within our cell-signalling and metabolic framework, as oxidative metabolism is not reduced alongside AMPK activation, potentially attenuating mTOR dysregulation, reducing insulin resistance, and restoring insulin signalling and the PI3K/AKT pathway.

Metformin has also been used in schizophrenia; however, they have typically been used alongside antipsychotics to reduce weight gain [464]. One review by Battini et al. [465] examined 19 RCTs on the effectiveness of metformin in reducing cognitive impairment and psychotic symptoms. They found a small positive benefit for metformin use compared to controls across cognitive impairment, and to a lesser extent, positive symptoms, despite the confounding effect of antipsychotic use. Glucagon-like peptide-1 receptor agonists (GLP-1s) have also been explored in schizophrenia, again typically to reduce the metabolic side effects of antipsychotic use; current evidence shows they are highly effective at treating metabolic outcomes, but show no improvements in psychiatric outcomes [466]. GLP-1 mimics the natural GLP-1 hormone, stimulating glucose-dependent insulin secretion and suppressing glucagon production [467]. Theoretically, GLP-1s could promote IRS-1 activity by recruiting IRS-1 to the insulin receptor and suppressing its degradation, thereby increasing PI3K/AKT activity [468]. However, current GLP-1 drugs are not believed to be able to cross the blood–brain barrier, and it is unclear how much of a counter-effect antipsychotic medication would have on their mechanisms of action [469]. Furthermore, given the mitochondrial and antioxidant damage associated prenatally with schizophrenia, research should look to developing drugs that target this early mechanism or focus on attenuating their risk during development, not once schizophrenia has reached its final stages.

Ketamine is also emerging as a treatment for depressive disorders. A recent systematic review and meta-analysis of ketamine and esketamine for depression suggested that these drugs are effective, rapidly acting antidepressants, particularly with treatment-resistant depression, with results showing reduced depressive symptom severity, rapid clinical improvements within hours, and reduced suicidal ideation [470]. Effects were similar in bipolar depression; however, there is less evidence available. Several other reviewers have theorised that this rapid antidepressant effect is not due to targeting monoamine (neurotransmitter) deficiencies, and instead suggest ketamine rapidly restores synaptic plasticity that has been impaired by chronic stress [471]. Interestingly, this rapid antidepressant effect has been suggested to result from ketamine acting on the BDNF, TrkB, PI3K/AKT, and mTOR pathways, thereby promoting rapid protein synthesis, synaptogenesis, and restoration of stress-induced synaptic deficits [471,472]. Ketamine in healthy populations has been shown to produce schizophrenia-like positive symptoms, negative symptoms, cognitive deficits, and thought disorders, which has led to ketamine becoming one of the major pharmacological models of schizophrenia [473]. Ketamine as a treatment for schizophrenia has been suggested to improve depressive and negative symptoms, but there is currently little evidence for its effectiveness in positive symptoms or enough evidence to conclude that it is an effective treatment [474]. This may suggest that exogenously correcting synaptic plasticity alone is insufficient to substantially improve schizophrenia.

Finally, current research into the ketogenic diet is showing promising results for treating schizophrenia, depression, and bipolar disorder; future research is warranted to establish exactly how the diet is impacting the underlying mechanisms and producing positive results [402]. Theoretically, the ketogenic diet seems to mimic several positive benefits of current treatment methods, while also bypassing glycolysis by supplying acetyl-CoA directly to the TCA cycle, enabling oxidative ATP production independent of insulin, while also reducing nutrient overload, improving insulin sensitivity, suppressing mTOR activity, restoring PI3K/AKT function, and promoting antioxidant defence, which mitigates several negative downstream effects. However, the current body of literature has generally assessed only insulin resistance and metabolic syndrome, and its findings could be strengthened by evaluating the ketogenic diet’s effectiveness in these disorders by assessing its effects on neurological function, oxidative metabolism, mitochondrial function, and the PI3K/AKT and mTOR pathways in patients with schizophrenia, depression and bipolar disorder.

## 9. Conclusions

This review builds on the emerging frameworks of broken bioenergetic and impaired dynamic metabolic flexibility in schizophrenia. This framework expands on the original theory’s by incorporating findings from recent research on insulin resistance, insulin signalling, and the PI3K/AKT, MEK/ERK, and mTOR signalling pathways.

These environmental sensing and energy-adaptation signalling pathways may mechanistically contribute to schizophrenia through a time-dependent and cell-type-specific process: initial insulin resistance may drive mTORC1 hyperactivation in neurons without a corresponding glycolytic shift, owing to their HK1-dominant, glycolysis-resistant profile, while promoting a glycolytic and inflammatory shift in HK2-permissive microglia. Sustained neuronal mTORC1 hyperactivity may in turn damage mitochondria by impairing mitophagy and biogenesis. This reduction in oxidative phosphorylation capacity, combined with insulin resistance limiting the glucose available for a compensatory glycolytic response, may leave neurons increasingly reliant on lactate as an alternative fuel source.

The benefit of this expanded theory is that it summarises the current available evidence into a holistic framework, incorporating recent metabolic evidence into current frameworks of schizophrenia without devaluing other competing theories. Furthermore, it provides a mechanistic theory for how insulin resistance and mitochondrial dysfunction contribute to schizophrenia, depression, and bipolar disorder. However, given its theoretical nature, significant research is needed to validate the framework. Nonetheless, it also provides several other future research routes that could improve current treatment methodologies for severe mental illnesses.

## Figures and Tables

**Figure 1:**
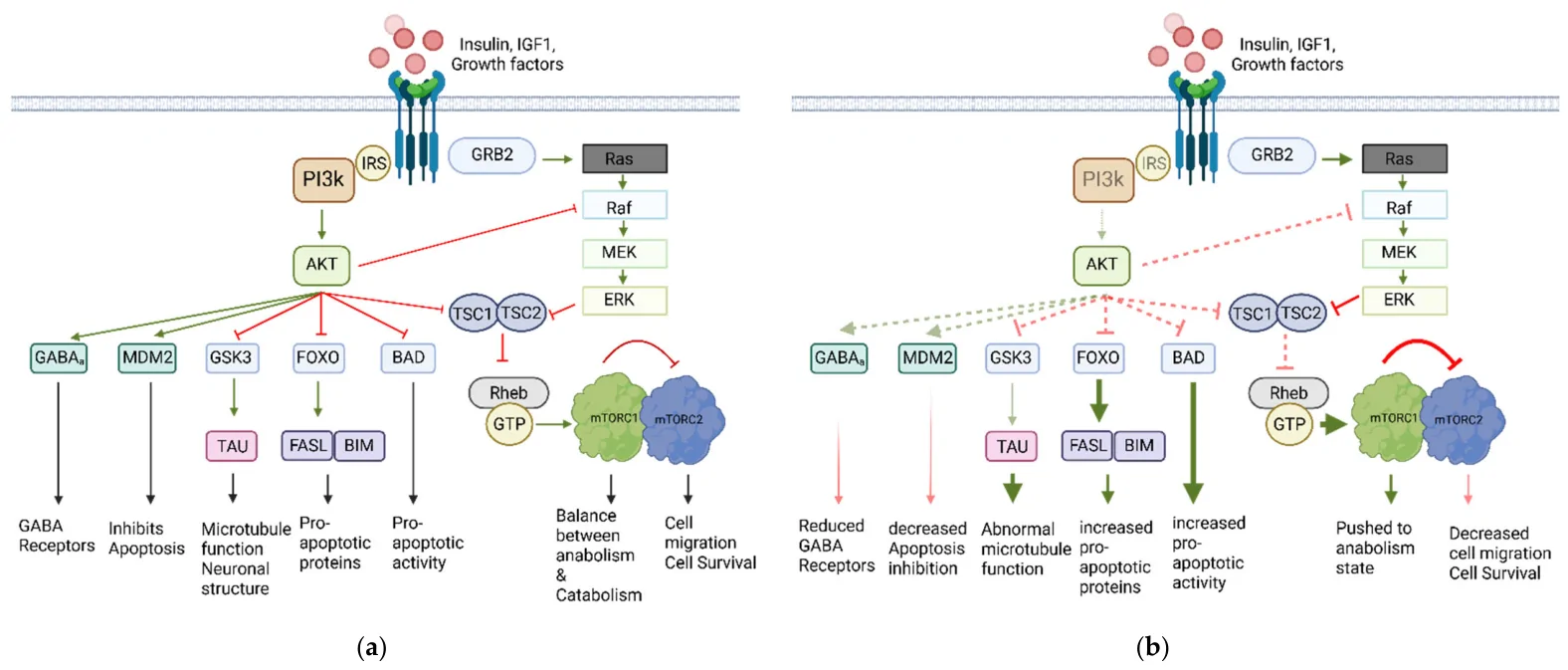
(**a**) PI3k/AKT signalling under healthy conditions. (**b**) Abnormalities in insulin signalling and insulin resistance result in decreased PI3K/AKT signalling. Decreased AKT activity leads to reduced activity of GABA_A_ and MDM2, and decreased inhibition of GSK3, FOXO, BAD, and TSC1/2. Uninhibited FOXO activates FASL and BIM. Reduced AKT expression should inhibit mTORC1 via TSC/RHEB-GTP; however, reduced crosstalk between AKT and RAF allows continued MEK/ERK (MAPK) signalling, ultimately activating mTORC1. Finally, uninhibited GSK3 increases TAU phosphorylation.

**Figure 2:**
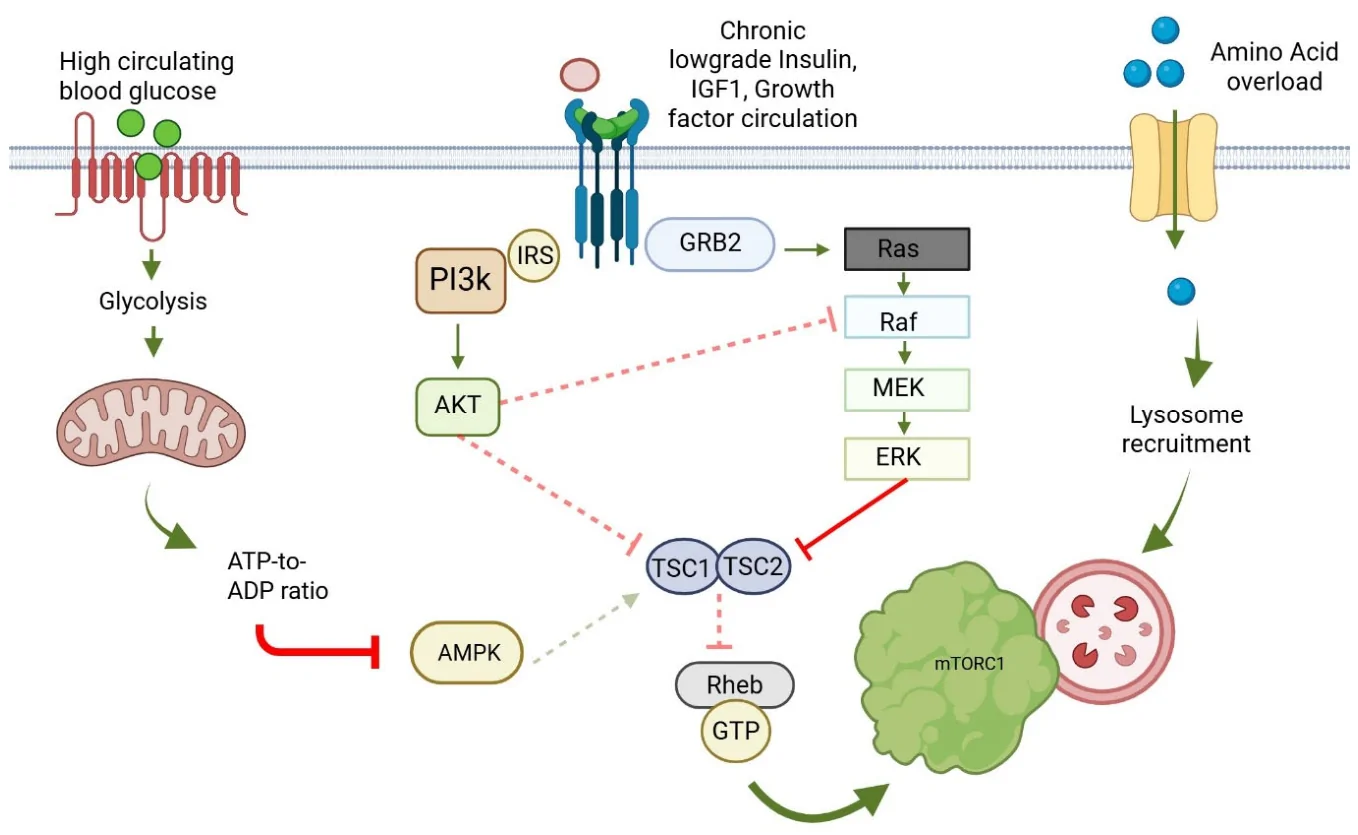
Abnormalities in mTOR function in IR conditions. A high ATP-to-ADP ratio inhibits AMPK, preventing activation of TSC1/TSC2. Chronic low-grade MEK/ERK activation inhibits TSC1/TSC2. TSC1/TSC2 remains inhibited, allowing Rheb to chronically activate mTORC1. Amino acid overload translocates mTORC1 to the lysosome, allowing full activation. Note—details in each step have been omitted for clarity.

**Figure 3:**
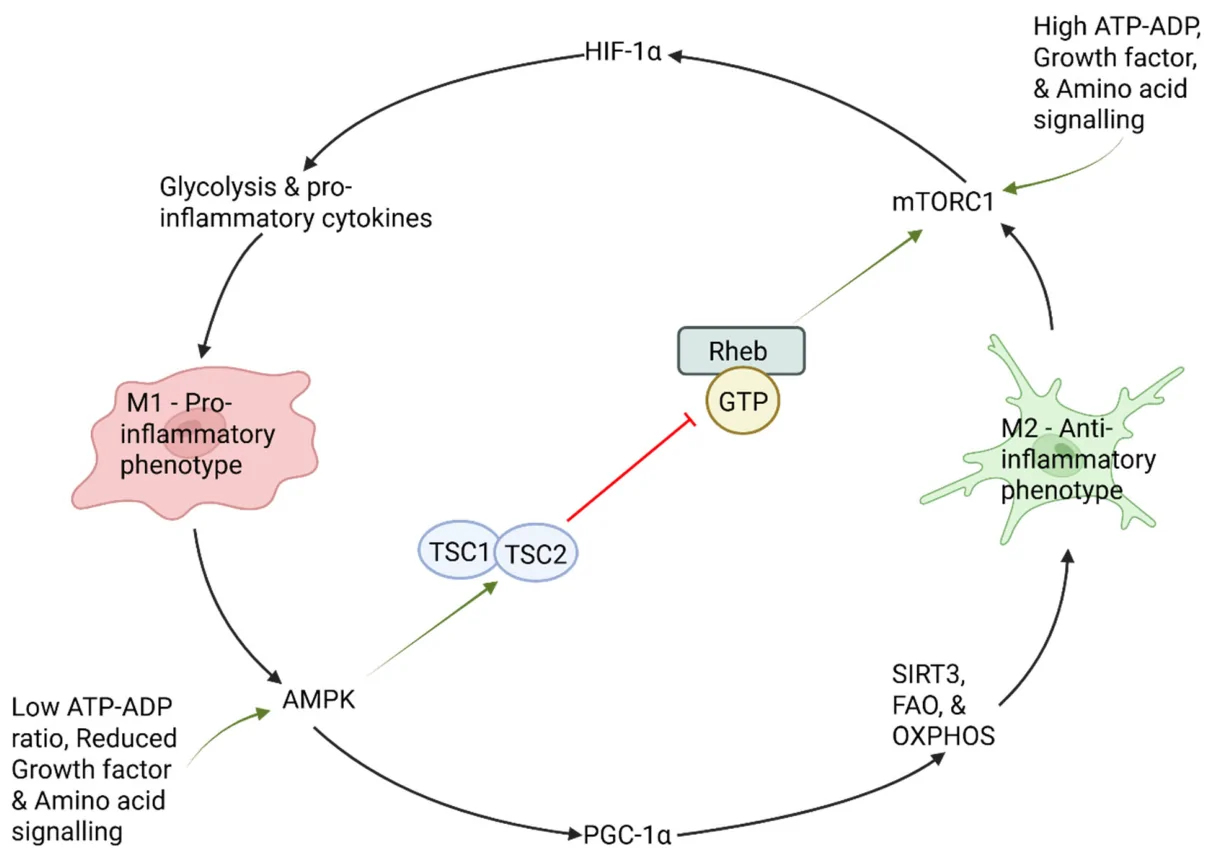
Theoretical mechanism by which metabolically opposed AMPK and mTORC1 regulate microglial phenotypes. AMPK is activated under low energy, alongside reduced growth factor and amino acid signalling, which activates TSC1/2, inhibiting Rheb from activating mTORC1. AMPK activates PGC-1α, which upregulates genes that promote SIRT3, FAO (fatty acid oxidation), and OXPHOS (oxidative metabolism), ultimately leading to increased mitochondrial biogenesis and activity, inflammation resolution, and improved tissue healing. On the other hand, high ATP/ADP, growth factor, and amino acid signalling activate mTORC1, which in turn upregulates HIF-1α. HIF-1α then upregulates glycolysis, leading to the creation of pro-inflammatory cytokines. Within our mechanistic framework, IR and nutrient overload lead to chronic hyperactivation of mTORC1, biassing microglia towards the M1—pro-inflammatory phenotype.

**Figure 4:**
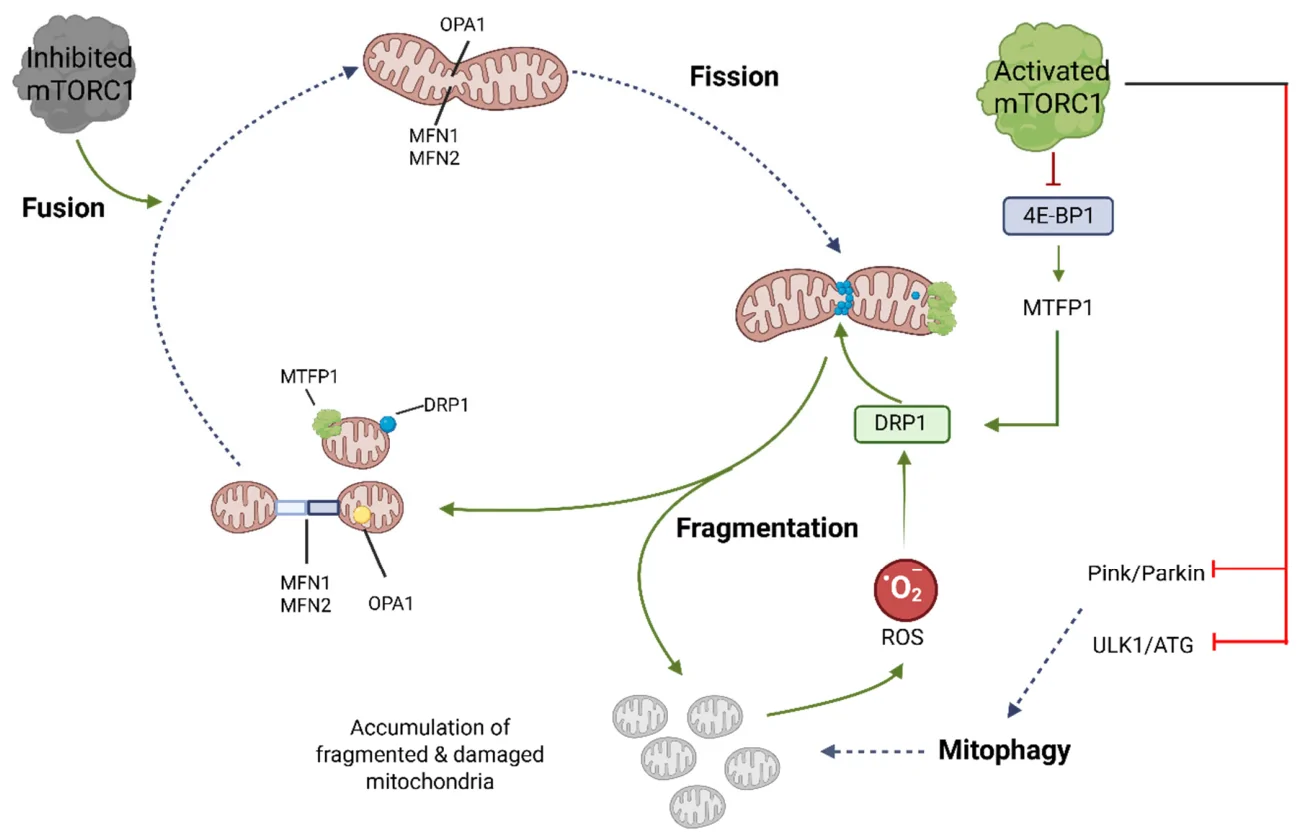
Theoretical mechanism by which mTORC1 regulates mitochondrial fusion/fission balance and mitophagy. Inhibited mTORC1 promotes fusion. Activated mTORC1 promotes fission by inhibiting 4E-BP1-mediated repression of MTFP1 translation. Similarly, activated mTORC1 inhibits PINK1 expression, Parkin translocation, and formation of the mitophagy-initiator complex ULK1/ATG. Hyperactive mTORC1 results in the accumulation of fragmented, damaged, and ROS-producing mitochondria. This increased ROS production then operates in a feed-forward loop, promoting fragmentation by increasing DRP1 activity.

**Table 1:** Overlap of key mechanistic components in depression and bipolar disorder.

	Depression	Bipolar Disorder
PI3K/AKT	Reduced PI3K/AKT expression and phosphorylation in humans and animal models. Increased PI3K/AKT signalling is associated with antidepressant effects [330].	Dysregulated. Postmortem studies report reduced PI3K/AKT activity [331]. Downstream abnormalities implicating dysregulation and altered mood states in humans and animal models [332,333].
mTOR	Significant reduction in mTOR and its downstream transcriptional targets in the prefrontal cortex in humans [334]. Weaker animal model evidence, with chronic stress showing depressive behaviour and reduced activity of mTOR and its targets in the PFC, hippocampus, and amygdala [335].	Reduced mTOR mRNA in the blood of drug-naïve patients in depressive episodes [336]. Reduced mTOR signalling in the PFC [331]. Animal models of mania show elevated mTOR activity, whereas human studies show depressive episodes are associated with downregulation [337].
GABA_A_ receptors and GABA function	Altered expression of GABAα receptors [338]. Reduced GABA compared to healthy controls; stronger reductions in treatment-resistant groups; some evidence of no reductions [339,340].	Altered expression of GABAα receptors [338,341]. GABAergic dysregulation and impaired inhibitory neurotransmission [339].
Dopamine	Reduced mesolimbic dopamine signalling, including in the ventral tegmental area (VTA), nucleus accumbens, and the PFC, with weaker evidence in the amygdala and hippocampus [342].	Increased striatal dopamine transporter (DAT) availability, leading to reduced mesolimbic (VTA and NA) dopamine in depressive states [343]. Reduced DAT, increased transmission, increased dopamine synthesis capability in manic states [344].
Glutamate	Impaired glutamatergic neurotransmission and altered glutamate cycling, most consistently implicated in the PFC, anterior cingulate cortex, and the hippocampus [345].	State-dependent glutamatergic dysregulation, with elevated cortical glutamate most consistently observed during mania, while depression demonstrates more heterogeneous abnormalities [346].
ER stress/CA^2+^ dysregulation	Increased ER stress and activation of the unfolded protein response (UPR) [347]. Altered intracellular Ca^2+^ signalling and disrupted ER Ca^2+^ homeostasis [348].	Increased ER stress and dysregulation of the UPR [349]. Altered intracellular Ca^2+^ signalling, disrupted ER Ca^2+^ homeostasis, and altered ER–mitochondrial Ca^2+^ signalling [350,351].
HIF-1α, VEGF, GLUT1/3, and glycolytic enzymes	Increased mRNA expression of HIF-1α, VEGF, GLUT1, PFKB3, PGK1, and LDHA in peripheral cells [352]. No difference in VEGF levels between controls [353].	Increased mRNA expression of HIF-1α, VEGF, and PFKB3 in peripheral cells in depressive states [352]. No difference in VEGF levels between controls [353].
OXPHOS	Impaired ETC activity, reduced OXPHOS, decreased ATP production, and increased oxidative stress [354,355]. Altered brain lactate metabolism [356].	Impaired OXPHOS, decreased ATP production, and increased oxidative stress [357]. Elevated brain lactate [140,358]. Emerging evidence suggests OXPHOS may be mood state dependent, lower in depression and higher in mania [327].
Inflammation	Increased microglial activation, increased microglial glycolysis and reduced OXPHOS; increased IL-1β, IL-6, TNF-α [359].	Increased microglial activation, IL-6, and TNF-α [360]. Limited direct evidence of microglial glycolysis and OXPHOS.
PGC-1α, SIRT1/3	Reduced PGC-1α activity/expression [355]. SIRT1 risk gene association and reduced SIRT1 in the periphery; limited evidence for SIRT3 [361].	Limited evidence for PGC-1α and SIRT1/3, with only speculative evidence surrounding mitochondrial physiology [362].
Mitochondrial dynamics/regulation	Increased fission, reduced fusion, impaired mitophagy, impaired biogenesis, increased DRP1, reduced expression/activity of MFN1/2 and OPA1, reduced Pink/Parkin signalling [355,363].	Impaired mitochondrial dynamics are implicated, with evidence of altered mitochondrial morphology and increased fragmentation [327]. Limited mechanistic evidence, with some showing reductions in MFN2, OPA1, and Pink/Parkin.
Antioxidants	Altered SOD and glutathione antioxidants, and decreased total antioxidant capacity have been suggested, but findings are often heterogeneous [364].	Impaired antioxidant defence, reduced glutathione and increased oxidative damage [365]. However, other antioxidant findings are more heterogeneous.

## Data Availability

No new data were created or analysed in this study. Data sharing is not applicable to this article.

## Abbreviations

The following abbreviations are used in this manuscript:

4E-BPs	Eukaryotic translation initiation factor 4E-binding proteins
5-HT2A	5-Hydroxytryptamine receptor 2A (serotonin receptor 2A)
AcAc	Acetoacetate
Acetyl-CoA	Acetyl coenzyme A
ADP	Adenosine diphosphate
AG	Aerobic glycolysis
AKT	AKT serine/threonine kinase
AMP	Adenosine monophosphate
AMPK	AMP-activated protein kinase
APC	Adenomatous polyposis coli protein (microtubule-associated scaffold protein)
ATG	Autophagy-related proteins
ATP	Adenosine triphosphate
BAD	BCL-2-associated agonist of cell death (pro-apoptotic protein)
BAK	BCL-2 antagonist/killer 1 (pro-apoptotic protein)
BAX	BCL-2-associated X protein (pro-apoptotic protein)
BBB	Blood–brain barrier
BCL-2	BCL-2 (anti-apoptotic protein)
BCL-XL	BCL-XL (anti-apoptotic protein)
BDNF	Brain-derived neurotrophic factor
BHB	β-Hydroxybutyrate
BIM	BCL-2-interacting mediator of cell death (pro-apoptotic protein)
BMAL1	Brain and muscle ARNT-like protein 1
BMI	Body mass index
CACNA1C	Calcium voltage-gated channel subunit alpha1 C
Ca^2+^	Calcium ion
Caspase-3	Caspase-3
CHR	Clinical high risk (for psychosis)
CLOCK	Circadian locomotor output cycles kaput
CNS	Central nervous system
CRMP	Collapsin response mediator protein
CSF	Cerebrospinal fluid
D2	Dopamine D2 receptor
DAT	Dopamine transporter
CRY	Cryptochrome (circadian clock protein)
DNA	Deoxyribonucleic acid
DRP1	Dynamin-related protein 1 (mitochondrial fission protein)
ER	Endoplasmic reticulum
ERK1/2	Extracellular signal-regulated kinases 1 and 2
ETC	Electron transport chain
FAO	Fatty acid oxidation
FASL	Fas ligand (pro-apoptotic ligand)
FEP	First-episode psychosis
FOXO	Forkhead box O transcription factors
FOXO3	Forkhead box O3 transcription factor
GABA	Gamma-aminobutyric acid
GABA_A_R	Gamma-aminobutyric acid type A receptor
GAP	GTPase-activating protein
GAPDH	Glyceraldehyde-3-phosphate dehydrogenase
GLP-1	Glucagon-like peptide-1
GLUT1	Glucose transporter 1
GLUT3	Glucose transporter 3
GRB2	Growth factor receptor-bound protein 2
GSK3	Glycogen synthase kinase-3
GWAS	Genome-wide association study
HbA1c	Glycated haemoglobin
HCAR2	Hydroxycarboxylic acid receptor 2
HIF	Hypoxia-inducible factor
HIF-1α	Hypoxia-inducible factor-1 alpha
HK1/2	Hexokinases 1 and 2
HK2	Hexokinase 2
HOMA-IR	Homeostatic model assessment of insulin resistance
HPA axis	Hypothalamic–pituitary–adrenal axis
IGF-1	Insulin-like growth factor 1
IL-1β	Interleukin-1 beta
IL-6	Interleukin-6
IL-8	Interleukin-8
IL-10	Interleukin-10
IL-18	Interleukin-18
IR	Insulin resistance
IRS1/2	Insulin receptor substrates 1 and 2
LDH	Lactate dehydrogenase
LDHA	Lactate dehydrogenase A
LTD	Long-term depression
MAD1L1	Mitotic arrest deficient-like 1
MAD2	Mitotic arrest deficient 2
MAPK	Mitogen-activated protein kinase
MCTs	Monocarboxylate transporters
MDM2	Mouse double minute 2 homologue (E3 ubiquitin ligase)
MEK	Mitogen-activated protein kinase
MFN1/2	Mitofusin 1 and 2 (mitochondrial fusion proteins)
miR-137	MicroRNA-137
MOMP	Mitochondrial outer membrane permeabilisation
MTFP1	Mitochondria fission process protein 1
mTOR	Mechanistic target of rapamycin
mtDNA	Mitochondrial DNA
Myc	MYC proto-oncogene (transcription factor)
NAD^+^	Nicotinamide adenine dinucleotide (oxidised form)
NADH	Nicotinamide adenine dinucleotide (reduced form)
NF-κB	Nuclear factor kappa B
NGF	Nerve growth factor
NMDA	N-Methyl-D-aspartate receptor
OPA1	Optic atrophy protein 1 (mitochondrial fusion protein)
OXPHOS	Oxidative phosphorylation
PANSS	Positive and Negative Syndrome Scale
Parkin	Parkin E3 ubiquitin protein ligase
PER	Period circadian regulator
PFC	Prefrontal cortex
PFK-1	Phosphofructokinase-1
PGC-1α	Peroxisome proliferator-activated receptor gamma coactivator-1 alpha
PGK1	Phosphoglycerate kinase 1
PHDs	Prolyl hydroxylase domain proteins
PI3K	Phosphoinositide 3-kinase
PINK1	PTEN-induced kinase 1 (mitophagy kinase)
PKC	Protein Kinase C
PKM2	Pyruvate inase M2
p53	Tumour suppressor protein p53
p63	Tumour suppressor protein p63
RAF	RAF serine/threonine protein kinase
RAS	Rat sarcoma (RAS) small GTPase
RCT	Randomised controlled trial
REDD1	Regulated in development and DNA damage responses 1
Rheb	Ras homologue enriched in brain
RNA	Ribonucleic acid
ROS	Reactive oxygen species
S6K1	Ribosomal protein S6 kinase beta-1
SERCA	Sarco/endoplasmic reticulum Ca^2+^-ATPase
Sestrin1/2	Sestrins 1 and 2 (stress-response proteins)
SGK	Serum- and glucocorticoid-regulated kinase
SHC	Src homology and collagen adaptor protein
SIRT1/3	Sirtuins 1 and 3
SNPs	Single nucleotide polymorphisms
SOD2	Superoxide dismutase 2
SOS	Son of sevenless
Tau	Microtubule-associated protein tau
TCA cycle	Tricarboxylic acid cycle
TCF4	Transcription factor 4
TGF-β	Transforming growth factor beta
TNF-α	Tumour necrosis factor alpha
TrkB	Tropomyosin receptor kinase B
TSC	Tuberous sclerosis complex
UCP2	Uncoupling protein 2
ULK1	UNC-51-like kinase 1 (autophagy-initiating kinase)
UPR	Unfolded protein response
VDCCs	Voltage-dependent calcium channels
VEGF	Vascular endothelial growth factor
VTA	Ventral tegmental area
